# Gender, Contraceptives and Individual Metabolic Predisposition Shape a Healthy Plasma Lipidome

**DOI:** 10.1038/srep27710

**Published:** 2016-06-14

**Authors:** Susanne Sales, Juergen Graessler, Sara Ciucci, Rania Al-Atrib, Terhi Vihervaara, Kai Schuhmann, Dimple Kauhanen, Marko Sysi-Aho, Stefan R. Bornstein, Marc Bickle, Carlo V. Cannistraci, Kim Ekroos, Andrej Shevchenko

**Affiliations:** 1 MPI of Molecular Cell Biology and Genetics, Pfotenhauerstrstraße 108, 01307 Dresden, Germany; 2 Department of Internal Medicine III, Medical Faculty Carl Gustav Carus, Technische Universität Dresden, Fetscherstraße 74, 01307 Dresden, Germany; 3 Biomedical Cybernetics Group, Biotechnology Center (BIOTEC), Technische Universität Dresden, Tatzberg 47/49, 01307 Dresden, Germany; 4 Lipotype GmbH, Tatzberg 47, 01307 Dresden, Germany; 5 Zora Biosciences Oy Biologinkuja 1, 02150 Espoo, Finland; 6 Division of Diabetes & Nutritional Sciences, King’s College Hospital, NHS Foundation Trust, London, SE5 9RS, UK

## Abstract

Lipidomics of human blood plasma is an emerging biomarker discovery approach that compares lipid profiles under pathological and physiologically normal conditions, but how a healthy lipidome varies within the population is poorly understood. By quantifying 281 molecular species from 27 major lipid classes in the plasma of 71 healthy young Caucasians whose 35 clinical blood test and anthropometric indices matched the medical norm, we provided a comprehensive, expandable and clinically relevant resource of reference molar concentrations of individual lipids. We established that gender is a major lipidomic factor, whose impact is strongly enhanced by hormonal contraceptives and mediated by sex hormone-binding globulin. In lipidomics epidemiological studies should avoid mixed-gender cohorts and females taking hormonal contraceptives should be considered as a separate sub-cohort. Within a gender-restricted cohort lipidomics revealed a compositional signature that indicates the predisposition towards an early development of metabolic syndrome in *ca*. 25% of healthy male individuals suggesting a healthy plasma lipidome as resource for early biomarker discovery.

Blood plasma analysis is a cornerstone of clinical chemistry. Plasma is an abundant, readily available clinical resource whose composition is reflective of basic merits of metabolism and homeostasis. It contains informative molecular markers of basic pathophysiological processes such as inflammation, atherosclerosis or metabolic syndrome, to mention only a few. A typical blood test to diagnose metabolic syndrome or type 2 diabetes mellitus may report more than 30 clinically relevant indices, however only four of them, total triacylglycerols (TAG), total cholesterol (Chol) and the cholesterol content in HDL and LDL fractions, are directly reflecting the status of lipid homeostasis.

Since recently, human blood plasma is being extensively studied by lipidomics (reviewed in^1^). An inter-laboratory effort spearheaded by the LIPID MAPS consortium quantified 588 individual lipids from 21 major lipid classes^2^. Other plasma lipidome studies pinpointed individual molecules or entire lipid classes whose abundance was specifically altered in obesity^3^, type 1^4^ and type 2^5^ diabetes, insulin resistance^6^, hypertension^7^, cardiovascular disease^8^,^9^, Alzheimer’s disease^10^ and schizophrenia^11^,^12^. Associating lipidome changes with diseases progression shed light on their molecular mechanisms and metabolic consequences and lead to the identification of promising biomarkers^13^, means of dietary intervention^14^, or tools for monitoring the efficacy of lipid homeostasis correction through therapeutic or surgical treatments^15^,^16^.

Clinical lipidomics is an emerging field (reviewed in^17^) and standard operation procedures for quantifying lipids in biofluids and biopsies, as well as general guidelines for recruiting representative patient cohorts are yet to be established. One common approach is to determine relative (fold) changes between the abundance of lipid species in samples from disease and control cohorts and use statistical corrections to adjust for differences in age, BMI or common comorbidities (reviewed in^18^,^19^). While this may foster the discovery of disease-specific biomarkers, it neither makes the results of independent studies comparable nor improves our understanding of how complex pathologies (*e.g*. metabolic syndrome) impact the whole lipidome. Nowadays, lipids can be quantified by different means of mass spectrometry (reviewed in^20^,^21^,^22^) and accurate measurements should afford consistent molar values. However, the concordance of lipid concentrations determined by mass spectrometry and by common methods of clinical chemistry has so far received little attention.

Plasma lipidome varies between healthy individuals of different ethnic origin and is influenced by circadian rhythm^23^ and diet^24^. Lipid metabolism is also gender-dependent (reviewed in^25^), however it remains unclear how the molecular composition of plasma lipidome is affected by gender and if it is influenced by the level of sex hormones^26^. While numerous epidemiological screens compared plasma lipidomes of healthy and sick individuals in population-wide cohorts (reviewed in^27^), no reference values of lipid concentrations and their natural biological variance were established.

We applied shotgun lipidomics and liquid chromatography tandem mass spectrometry (LC-MS/MS) to quantify the molar concentrations of 281 molecules from 27 major lipid classes in the plasma lipidome of 36 male and 35 female healthy young Caucasians. We established that gender is a major lipidomic factor that is independent of major clinical and anthropometric indices and whose impact is strongly enhanced by hormonal contraceptive medication in females. Within a gender-restricted group, lipidomics revealed compositional trends indicating metabolic syndrome predisposition in currently healthy individuals.

## Results

### Quantitative differences between male and female healthy plasma lipidomes

**Figure 1:**
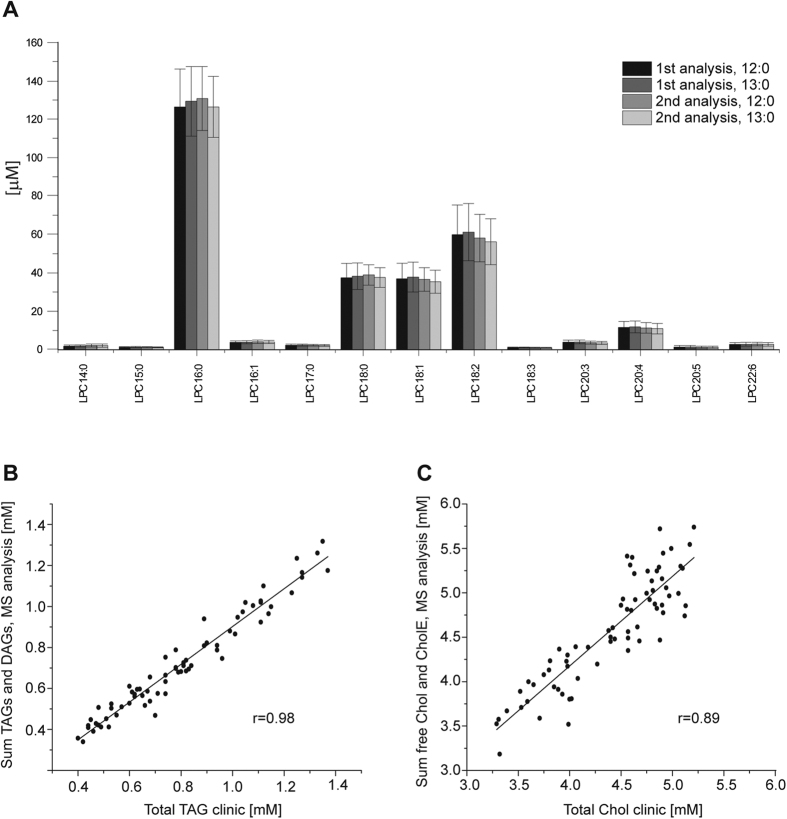
Absolute quantification of lipids is reproducible and corroborates common clinical indices. (**A**) Concentrations [μM] of LPC species in male plasma (*n* = 36) quantified in two independent analyses, each with two internal standards (LPC 12:0 and LPC 13:0). Data are mean ± SD. **(B)** Total TAG and (**C**) total Chol determined by shotgun mass spectrometry and clinical chemistry (*n* = 71). Data are the mean ± SD.

We used shotgun mass spectrometry and LC-MS/MS to determine absolute (molar) concentrations of 281 lipids from 27 major lipid classes. The accuracy and consistency of the lipid quantification was validated in two ways. First, we compared lipid concentrations determined in two independent series of experiments performed with a time gap of two months and each time using two independent internal standards for each lipid class (Fig. 1A). Second, for each member of the study cohort we summed up the concentrations of glycerolipids (48 TAG and 12 diacylglycerol (DAG) species) and compared it with the total concentration of TAG determined by the clinical blood test. In the same way, we summed up the concentration of free Chol and 15 cholesterol ester (CE) species and compared it with the cholesterol concentration from the blood test. Molar concentrations of glycerolipids and cholesterol determined by mass spectrometry and by clinical chemistry were concordant. On average, the difference was −10.4% (r = 0.98) for TAG (Fig. 1B) and 4.1% (r = 0.89) for Chol (Fig. 1C).

Next, we assembled a representative anthropometrically homogenous cohort of locally recruited medical students consisting of 36 male and 35 female Caucasians under the age of 33 years. According to the collected anamnesis, each individual had a clean medical record, received no pharmacological treatment at the time of investigation and further examination by a physician revealed no factors commonly comorbid with metabolic disorders. For each individual all 35 clinical indices reported by the blood test, blood pressure and anthropometric indices, including body mass index (BMI) and waist-to-hip ratio (WHO) were within ranges generally accepted as a gender-dependent medical norm. As expected, mean values of these indices also differed between the male and female sub-cohorts. For example, mean BMI differed by 1.2-fold between males and females. However, within each sub-cohort these values varied by less than 10% (Supplementary Tables S1 and S2) suggesting their anthropometric and physiological homogeneity. Therefore, stringent recruitment criteria and focus on young individuals with a clinically documented health status alleviated the need to recruit a larger study cohort without compromising the interpretation confidence.

**Figure 2:**
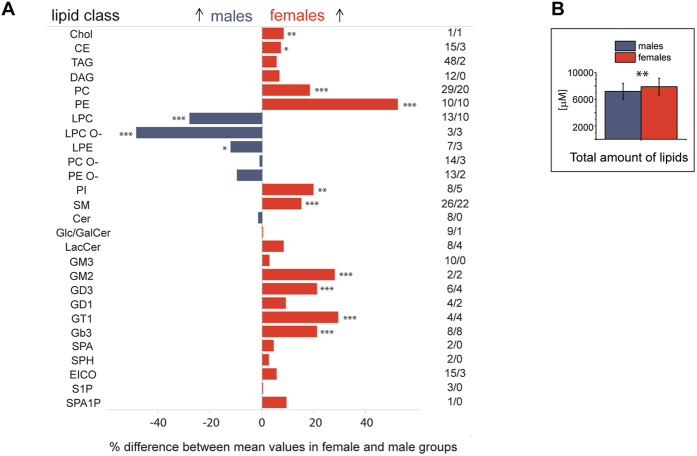
Plasma lipidomes of males and females are different. (**A**) Differences in % in the total concentration of lipid classes in male and female plasma. Lipid classes are at the left hand side; numbers at the right hand side indicate the total number of quantified species in each class/number of species that are significantly (*p* < 0.01) changed between males and females. Arrows indicate a higher concentration within the cohort. (**B**) Total concentration of all lipids for male (*n* = 36) and female (*n* = 35) sub-cohorts. *p < 0.05; **p < 0.01; ***p < 0.001 (Mann-Whitney nonparametric test with Benjamini-Hochberg correction). Data are the mean ± SD.

The analysis of healthy plasma lipidomes by mass spectrometry revealed pronounced differences in their molecular composition (Supplementary Table S2). Out of 281 quantified lipids, the abundance of 112 species was significantly (*p* < 0.01) different (Fig. 2A). Consistently with the clinical blood test, the total lipid content (Fig. 2B) and the total abundance of 21 out of 27 lipid classes were elevated in females with the notable exception of lyso-lipids, ether lipids and ceramides (Cer) (Fig. 2A). The magnitude of concentration differences was lipid class-dependent. It was as high as 50% for phosphatidylethanolamines (PE) and lyso-phosphatidylcholine ethers (LPC O-); at the same time, the difference in sphingomyelin (SM) concentration was small (<20%) yet highly significant (*p* < 0.001). Cer, PE O-, PC O- and, interestingly, also DAG and TAG, were least affected by gender (Fig. 2A).

### Hormonal contraceptives strongly impact the female plasma lipidome

**Figure 3:**
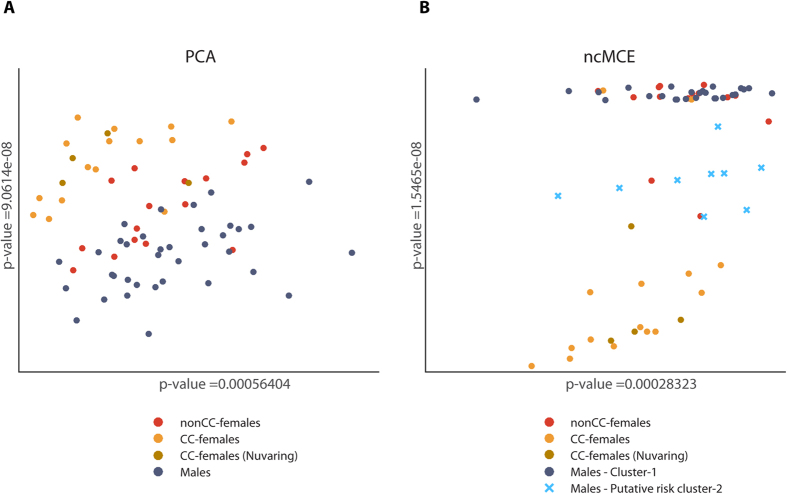
Unsupervised multivariate analyses by PCA and ncMCE reveal compositionally distinct lipidome clusters. Both PCA (**A**) and ncMCE (**B**) reveal a cluster of lipidomes of females taking hormonal contraceptives (CC-females, *n* = 19) (*p* < 0.001). CC-females (Nuvaring) indicate individuals using vaginal rings while other CC-females were taking pills. Among male lipidomes, ncMCE (**B**) distinguishes Cluster I (*n* = 27) and Cluster II (*n* = 9).

We wondered whether differences between male and female plasma lipidomes were common to all or to only some members of the cohort? We therefore analysed the lipidomics dataset by unsupervised principal component analysis (PCA) (Fig. 3A) and by non-centred Minimum Curvilinear Embedding (ncMCE)^28^,^29^ analysis (Fig. 3B). These methods rely on different, yet complementary computational principles^30^. PCA reflects relations based on the sample variance in the high-dimensional space, whereas ncMCE captures relations based on the hierarchical organization of the samples. Although the distribution of data points (here reflecting full individual lipidomes) may look different, the similarity of their clustering patterns provides an independent evidence of their compositional likeness.

Within the female cohort PCA and ncMCE distinguished two partially intermingling sub-groups reflecting the use of hormonal contraceptives (CC) by 19 participants (CC-females). Their lipidomes were separated with high statistical confidence from the lipidomes of males and females not taking CC (nonCC-females), whereas lipidomes of nonCC-females and males partially overlapped (Fig. 3A,B). At the same time, we observed no clear impact of the type of CC-medication. The 4 females who used vaginal rings (Nuvaring) did not group separately from females taking CC as pills.

### CC-dependent and CC-independent gender-related differences

We reasoned that both the intake of CC and inherent gender-related metabolism could be responsible for the observed compositional differences.

**Figure 4:**
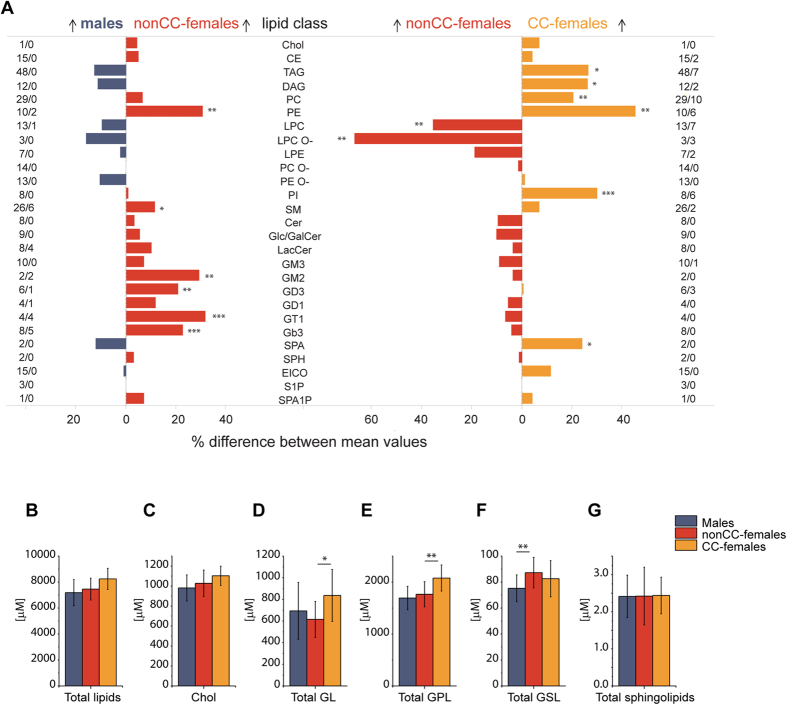
Plasma lipidomes of males, nonCC-, and CC-females differ in many aspects. (**A**) Relative differences (in %) in the concentration of lipid classes in males (n = 36) *vs* nonCC-females (n = 16) and in nonCC- *vs* CC-females (n = 19). Lipid classes are in the middle; numbers at the right and left hand side indicate the total number of detected species in the class/number of species that are significantly (*p* < 0.01) changed. Arrows indicate higher amount in the respective sub-cohort. (**B**) Total concentration of lipids; (**C**) total concentration of Chol; (**D**) total concentration of GL; (**E**) total concentration of GPL; (**F**) total concentration of glycosphingolipids (GSL); (**G**) total concentration of sphingolipids. *p < 0.05; **p < 0.01; ***p < 0.001 (Mann-Whitney nonparametric test with Benjamini-Hochberg correction). Data are the mean ± SD.

To delineate the basal differences we first compared the lipidomes of nonCC-females and males. Then, by comparing nonCC-females against CC-females, we identified the differences that arose from or were enhanced by pharmacological interference (Fig. 4).

A hallmark difference between the lipidomes of males and nonCC-females was the enrichment of glycosphingolipids (GSL) and SM (Fig. 4A; Supplementary Figs S1 and S2, Supplementary Table S3) in nonCC-females. Four out of 8 classes of GSL were significantly higher (*p* < 0.01) and also stood out in the comparison between males *vs* all females (Fig. 2A). At the same time, among GPL the abundance of PE was significantly increased in females. Interestingly, glycerolipid (GL) concentrations were hardly influenced by gender. No DAG or TAG species was significantly different (Supplementary Table S3).

**Figure 5:**
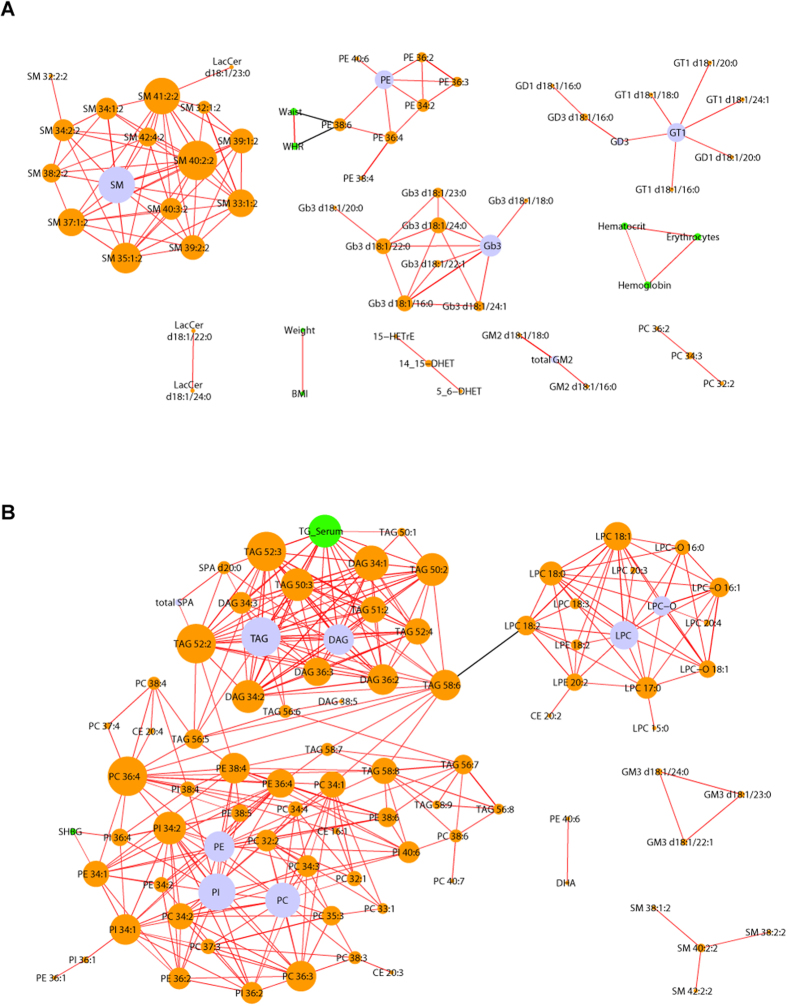
Correlation network of clinical and lipidomic values of males *vs* nonCC-females (**A**) and CC-females *vs* nonCC-females (**B**). The green nodes are the significantly changed clinical and anthropometric indices; the orange and blue nodes are significantly changed lipid species and classes, respectively. Red edges stand for positive and black edges for negative correlation. The size of nodes reflects the number of its significant correlations.

Mean values of anthropometric (BMI, WHR, body weight) and clinical (blood pressure, LDL, HDL) indices differ between male and female sub-cohorts (Supplementary Fig. S3, Supplementary Table S1) and we wondered if they were also covariate to gender discriminative lipids? Do lipid concentrations only reflect apparent anthropometric differences, or are they neatly associated with differently regulated lipid metabolism? To answer this question we built a correlation network representing significant associations between gender-discriminative abundances of lipid classes and individual species with anthropometric and clinical indices (Fig. 5A). We observed that clinical indices only associated with each other within a few closed clusters with no evidence of covariate relationship to gender discriminating lipids.

The comparison of lipidomes of nonCC-females against CC-females, on the other hand, suggested that hormonal medication shifted the female lipidome composition further away from the male lipidome. However, it did not markedly affect GSL concentrations (Figs 2A and 4A). At the same time, the impact of CC on GPL and GL classes was remarkable: concentrations of 5 out of 8 GPL classes were significantly different (*p* < 0.01). CC increased plasma concentrations of phosphatidylcholines (PC), PE, and phosphatidylinositols (PI), and lowered the concentration of lyso-lipids (LPC and LPC O-). Concentrations of PE and PC were already higher in the lipidome of nonCC-females compared to males (Fig. 2A), and they were even stronger enriched in CC-females (Fig. 4A). The same trend was observed for lipid classes enriched in males. Concentrations of LPC, LPC O- and LPE were lower in nonCC-females than in males, and they were even stronger depleted from the plasma of CC-female (Fig. 4A).

If compared to males and nonCC-females, plasma of CC-females accumulated more lipids in total (Fig. 4B), including Chol, GL and GPL (Fig. 4C–E, respectively). In contrast, plasma of nonCC-females was enriched in GSL compared to males and the difference remained unaffected (and even slightly reduced) by CC (Fig. 4F). Concomitantly, neither sphingolipids (Fig. 4G), nor ether-lipids (PC O- and PE O-), Cer, and eicosanoids were affected by gender alone or by CC (Fig. 4A). These compositional relationships were also reflected in the association network (Fig. 5B). The elevated content of TAG (according to mass spectrometry and clinical chemistry) correlated positively with unsaturated GPL and inversely with lyso-lipids, while showing no association with anthropometric and clinical indices, with the interesting exception of SHBG.

Altogether, gender and CC impact plasma lipidomes in different ways. While the concentration of GPL and GL was strongly influenced both by gender and CC (Fig. 4D,E), the latter did not affect the concentration of GSL (Fig. 4F). Overall, CC medication further contrasted the native gender-related differences in lipid metabolism. Lipids already enriched in nonCC-females plasma are becoming even more abundant, while lipids depleted in nonCC-females plasma are depleted to an even larger extent.

What biochemical mechanisms could underlie the impact of CC on the lipidome? We noticed that intake of CC reduced the concentration of endogenous estradiol by *ca*. 3-fold (Supplementary Table S2). This might decrease the expression of the phosphatidylethanolamine-*N*-methyltransferase (PEMT) and, in turn, decelerate the conversion of polyunsaturated PE (such as PE 38:6 and PE 40:6) into corresponding PC^31^,^32^ (Supplementary Fig. S4). Although the comparison of plasma of CC- and nonCC-females shows trends consistent with this notion (Supplementary Fig. S4), the variation of levels of endogenous sex hormones, such as estradiol in nonCC-females or (free-) testosterone in males, did not translate into significant and consistent perturbation of the plasma lipidome composition (Supplementary Table S4). It also showed no association with both gender-discriminating lipids (Fig. 5A) and lipids strongly affected by CC (Fig. 5B). Concomitantly, the absence of strong correlation with the levels of other reproductive hormones in females (luteinizing hormone, thyroid-stimulating hormone, and follicle-stimulating hormone) indicates that the menstrual cycle might not be affecting the plasma lipidome composition.

**Figure 6:**
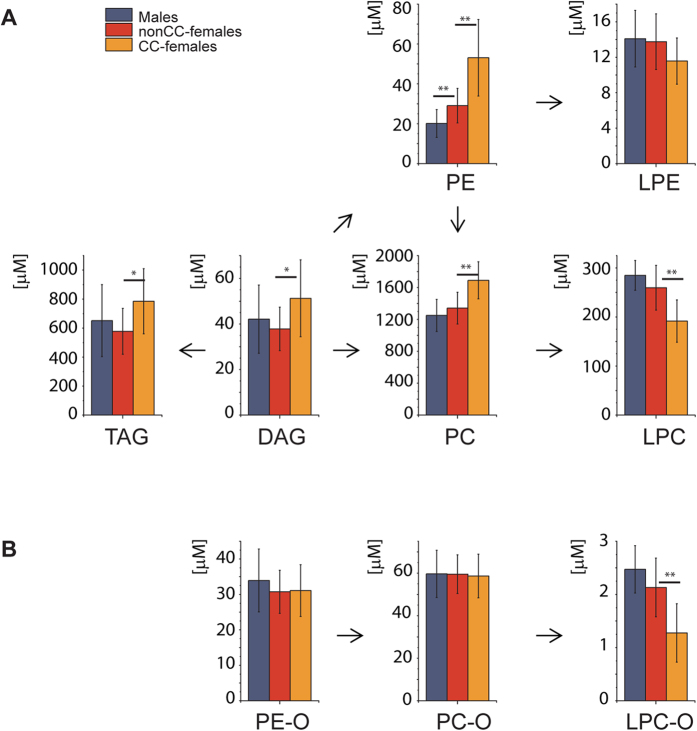
Contraceptives affect concentrations of GL and GPL, but not GSL. (**A**) Concentrations of some GL and GPL classes in males (*n* = 36), nonCC-females (*n* = 16) and CC-females (*n* = 19). (**B**) Concentrations of ether lipids and (**C**) GSL in the same cohort. Arrows indicate major pathways of lipids biosynthetic conversion. *p < 0.05; **p < 0.01; ***p < 0.001 (Mann-Whitney nonparametric test with Benjamini-Hochberg correction). Data are the mean ± SD.

Interestingly, CC-intake reduced the level of lyso-lipids, including LPC O-. LPC are inflammation responsive molecules (reviewed in^33^), whose content is reduced in plasma of patients suffering from *e.g*. liver inflammation. This corroborates with significantly (*p* < 0.001) elevated levels of the generic inflammation marker, C-reactive protein (hsCRP), in CC-females plasma (Supplementary Fig. S3), consistently with previous reports^34^,^35^. Conceivably, lower concentrations of lyso-lipids in the plasma of CC-females together with higher hsCRP suggests that low-grade inflammation induced by CC-intake might “stress” hepatocytes and enhance lipid biosynthesis (Fig. 6A,B).

### Concentration of sex hormone-binding globulin is elevated in the plasma of CC-females

Sex hormone-binding globulin (SHBG) is a plasma glycoprotein produced and secreted by the liver that regulates the bioavailability of sex hormones in circulation (reviewed in^36^). A lower plasma concentration of SHBG correlates with a higher risk for developing metabolic syndrome, type-2 diabetes, and cardiovascular disease. SHBG was also regarded as a major determinant of lipid plasma profile. This notion, however, was only supported by comparative analyses of common clinical indices of lipid metabolism, such as total concentration of TAG and HDL-cholesterol^37^.

**Figure 7:**
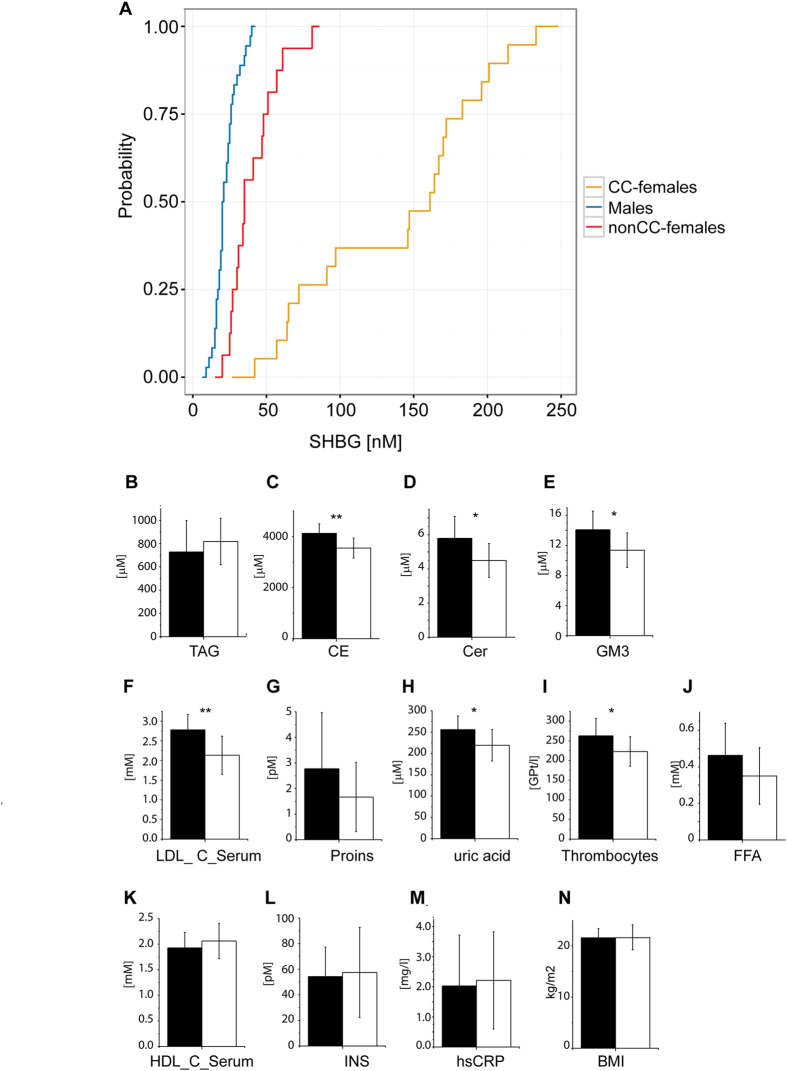
Elevated concentration of SHBG in CC-females favourably impacts both plasma lipidome composition and clinical indices related to lipid metabolism. (**A**) Cumulative distribution of the SHBG concentration in males (*n* = 36); nonCC-females (*n* = 16); CC-females (*n* = 19). (**B–N**) Concentrations of some lipid classes (**B–E**) and clinical indices (**F–N**) in CC-females with low (SHBG < 130 nM; *n* = 7; filled bars) and high (SHBG > 130 nM; *n* = 12; unfilled bars) SHBG levels. *p < 0.05; **p < 0.01; ***p < 0.001 (Mann-Whitney nonparametric test). Data are the mean ± SD.

We observed that SHBG concentrations were strongly elevated in CC-females compared to both nonCC-females and males (Fig. 7A). Interestingly, in respect to SHBG CC-females responded to CC in a very different way. SHBG concentrations exceeded 130 nM in a major fraction (12 out of the total of 19) of CC-females. Within this group, the average SHBG concentration was 2.5-fold higher compared to other CC-females (*n* = 7) and 4.5-fold higher compared to nonCC-females (*n* = 16). The increase of SHBG concentrations could not be associated with a specific type of CC and seemed to exclusively depend on the individual response.

We then asked if the difference in SHBG concentration is associated with a specific lipid profile or trend in clinical indices related to lipid metabolism? We therefore considered the two sub-groups of CC-females having SHBG concentration above (*n* = 12) and below (*n* = 7) an arbitrary threshold of 130 nM (Fig. 7A). Individuals from the “high SHBG” sub-group had lower concentrations of CE, Cer, and GM3. The same group also had lower concentrations of LDL, proinsulin, uric acid, thrombocytes, and free fatty acids (FFA) (Fig. 7F–J), while HDL, insulin and hsCRP were slightly increased (Fig. 7K–M) and there was no difference in BMI (Fig. 7N).

Taken together, CC could lead to a significant increase of SHBG in plasma, while the response was strongly individual and driven by a yet unknown factor. Higher SHBG concentration might decrease the risk of metabolic syndrome since several lipid classes (*e.g*. Cer, CE, GM3) and clinical parameters typically associated with its development were reduced^6^,^38^.

### Lipidome of healthy plasma revealed early signs of perturbed lipid metabolism

Statistical analyses indicated that lipidomes of both males and nonCC-females were compositionally heterogeneous (Fig. 3B). We therefore wondered if this heterogeneity only reflected an inherent biological variability between individual lipidomes, or was due to intermingling of compositionally distinct sub-groups? Since our study encompassed only 16 nonCC-females, here we only considered the compositional differences within the larger male cohort (*n* = 36).

**Figure 8:**
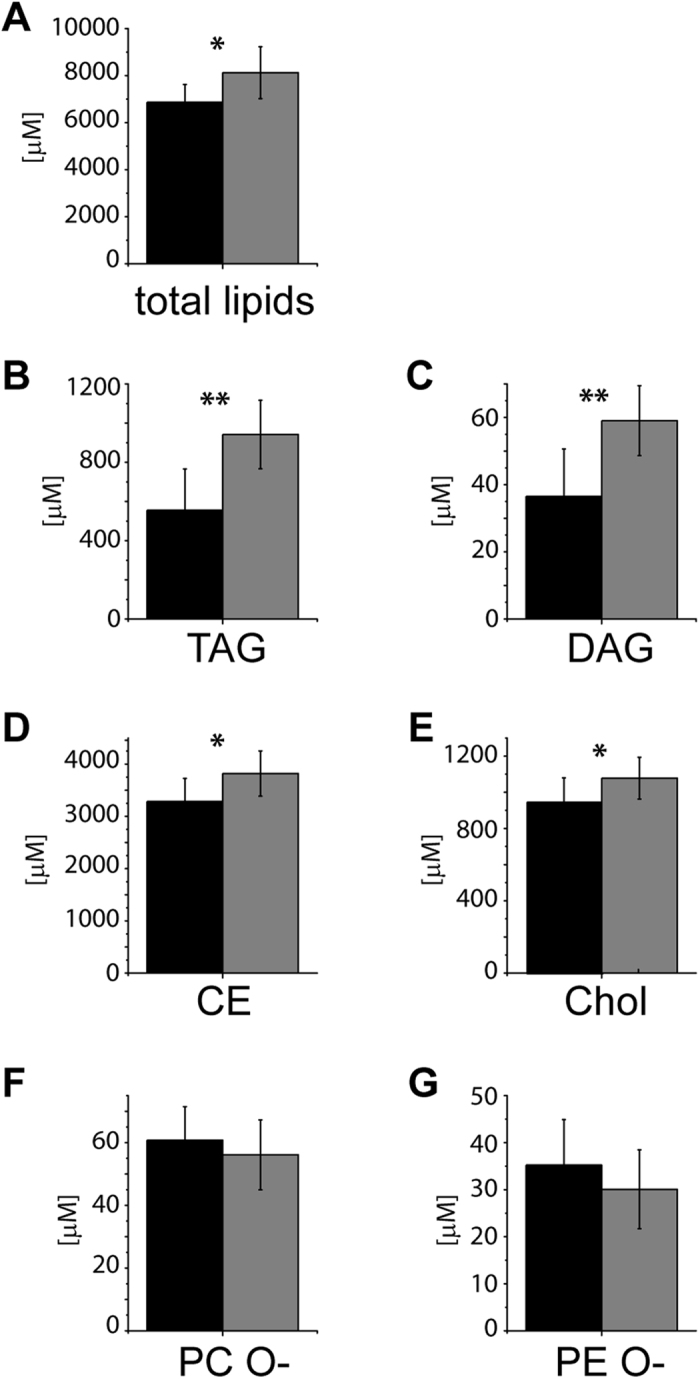
Lipidomes of the Cluster II members show early trends towards dyslipidemic metabolism. Comparison of concentrations of lipid classes in Cluster I (black bars; *n* = 27) and Cluster II (grey bars; *n* = 9). (**A**) total concentration of lipids; (**B**) TAG; (**C**) DAG; (**D**) CE; (**E**) Chol; (**F**) PC O-; (**G**) PE O-. *p < 0.05; **p < 0.01; ***p < 0.001 (Mann-Whitney nonparametric test with Benjamini-Hochberg correction). Data are the mean ± SD.

ncMCE (Fig. 3B) split the male cohort into two clusters termed Cluster I (*n* = 27) and Cluster II (*n* = 9). In comparison to Cluster I, plasma of Cluster II members contained *ca* 20% more lipids in total (Fig. 8A) along with particularly strong enrichment of TAG (77%) and DAG (63%) and moderate increase of CE and Chol (15%) (Fig. 8B–E). Concentration of other lipid classes increased to a variable extent with a clear tendency towards enrichment of species with unsaturated fatty acid moieties among glycero- (TAG; short and middle chain length DAG) and glycerophospholipids (PC, PI). The abundance of lyso-lipids and ceramides was practically unchanged (Supplementary Tables S2 and S3). Interestingly, the mean abundance of the ether lipids (PC O- and PE O-) (Fig. 8F,G) was reduced in Cluster II compared to Cluster I, consistently with the trend that was previously observed in hypertensive patients^7^. We reasoned that the lipidome of Cluster II might already show early indication of dyslipidemia or other manifestations of metabolic syndrome, such as hypertension in otherwise healthy individuals.

We next asked if anthropometric and clinical indices of members of Cluster II might be having a similar trend towards worsening lipid homeostasis, albeit their absolute values still remained within the normal range.

**Figure 9:**
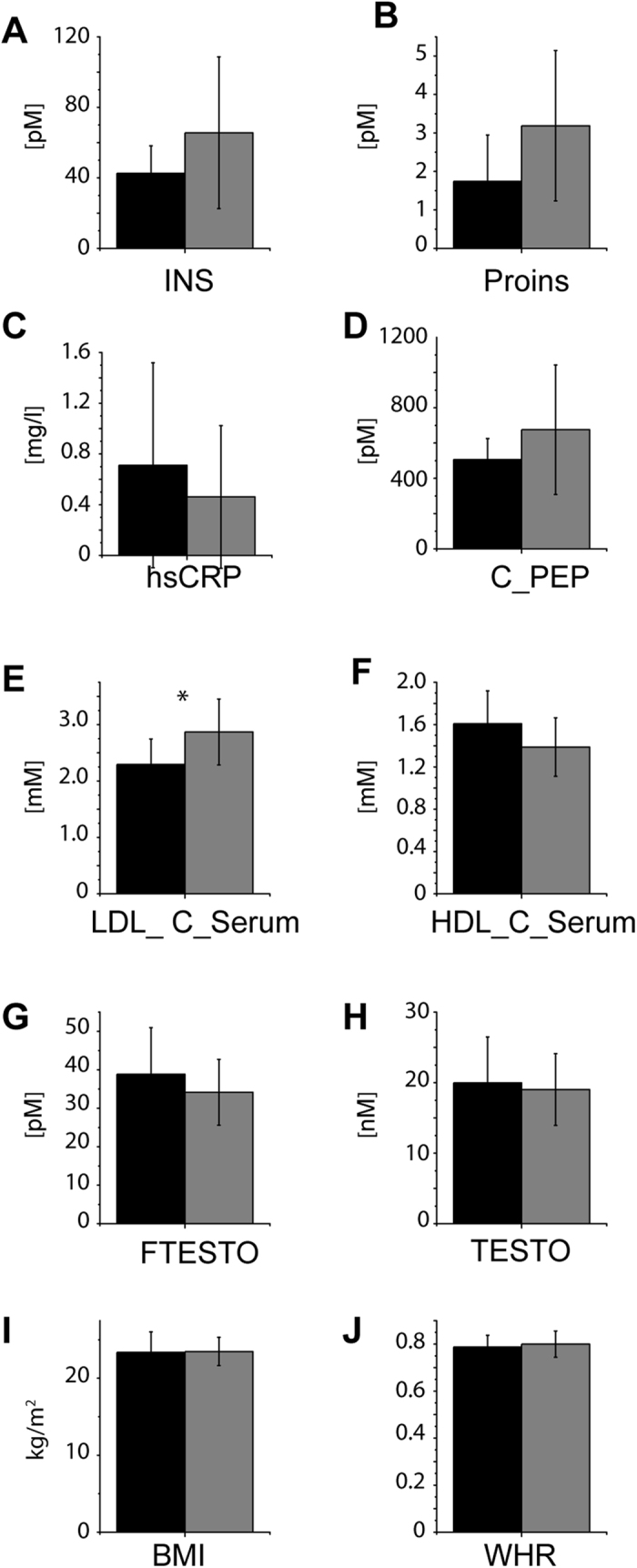
Clinical indices of the Cluster II members corroborate the dyslipidemic metabolism trend uncovered by lipidomics. Comparison of lipid metabolism-related clinical indices of the members of Cluster I (black bars; *n* = 27) and Cluster II (grey bars; *n* = 9). (**A**) insulin (INS); (**B**) proinsulin (Proins); (**C**) High-sensitive C-reactive protein (hsCRP); (**D**) C-peptide (C_PEP); (**E**) LDL concentration in serum; (**F**) HDL concentration in serum, (**G**) free testosterone (FTESTO); (**H**) testosterone (TESTO); (**I**) BMI; (**J**) waist to hip ratio (WHR). *p < 0.05; **p < 0.01; ***p < 0.001 (Mann-Whitney nonparametric test with Benjamini-Hochberg correction). Data are the mean ± SD.

Indeed, indices significant for metabolic syndrome were unfavourably altered in members of Cluster II: increased concentration of insulin, proinsulin and C-peptide, along with higher LDL and lower HDL, testosterone and free testosterone (Fig. 9A,B and D–H). At the same time, common anthropometrical indices (BMI, WHR) (Fig. 9I,J), and other general indices of homeostasis and metabolism (Supplementary Tables S2 and S3) remained unchanged. Interestingly, the inflammation related index hsCRP (Fig. 9C) was substantially reduced in Cluster II, although the major eicosanoids 12-HEPE and 12-HETE were both increased (Supplementary Tables S2 and S3).

TAG and DAG strongly differed between the two clusters (Fig. 8A,B). However, selecting the equivalent (*n* = 9) number of individuals with the highest absolute levels of TAG did not reproduce Cluster II (only 6 from 9 subjects were common) and lead to less pronounced differences between clinical indices (Supplementary Table S2). Although 8 out of 36 males (19.4%) had a BMI between 25.0 and 29.9 kg/m^2^ (overweight) only one of them was in Cluster II. Hence, monitoring the level of GL or BMI alone, either by clinical analyses or mass spectrometry, could not reveal perturbed metabolism trends.

We therefore concluded that, within the male cohort, lipidomics identified two sub-groups of otherwise healthy individuals. One subgroup (Cluster II; *n* = 9) showed trends that were reminiscent of those common to developed metabolic syndrome.

We observed similar, yet less pronounced, trends also within the nonCC-female cohort. Statistical analyses defined a small cluster (*n* = 5) of lipidomes having decreased concentration of ether- lipids, yet in contrast to males, their TAG and CE levels were practically unchanged. These individuals showed similar trends towards worsening metabolic indices compared to other nonCC-females, however the small number of selected individuals did not allow us to reach a definitive conclusion.

## Discussion

We established the reference values and biological variance of molar concentrations of individual plasma lipids for an age-restricted, ethnically and anthropometrically homogeneous cohort of male and female individuals having no noticeable health and, particularly, metabolism abnormalities. The health status of each study subject was established by 35 indices of a clinical blood test, anthropometric indices, anamnesis and the examination by a physician. Stringent recruitment of healthy individuals alleviated the need to adjust for common comorbidities and allowed us to reduce the cohort size without compromising the interpretation confidence. This notion corroborates the recent report by Begum *et al*. that showed that statistically confident comparison of healthy lipidomes is also possible using very small study cohorts^24^. We underscore that this specially recruited healthy, young, ethnically and socially homogeneous cohort does not reflect the diversity of an “average” local population. The reported reference values may therefore serve as a resource to explore the “multidimensionality” of the lipidome variability under medical, ethnical and social contexts.

Our interpretation of lipidome compositions solely relied upon molar concentrations of lipid molecules consistently detected throughout the cohort. While this was hardly possible in the past, now a wide and constantly expanding palette of high quality lipid standards is becoming available^39^. The reference values we reported here could be directly compared with the results of other studies, independently of their design or employed analytical methods. In this way, quantification discrepancies could be spotted and addressed through targeted validation procedures, including the use of multiple lipid standards and independent determinations by conventional methods of clinical chemistry (Fig. 1). We argue that reporting absolute quantities of lipids (rather than their fold changes relative to some arbitrary baseline values) should be generally adopted as a prerequisite clause for clinical lipidomics studies.

Our study revealed two major trends in gender-related lipidome differences: the content of GSL and SM (but not Cer) was significantly different between males *vs* nonCC-females, while they were not (or considerably less) different within the female-restricted cohort. Strong gender-related differences in Gb3 levels were previously observed in mice tissues^40^ and were thought to reflect metabolism differences associated with Fabry disease. However, we found that in human plasma these differences spanned the entire pool of gangliosides, lactosyl- and glucosylceramides, *i.e*. they were independent of both glycosylation type and exact structure of the sphingosine backbone. The second trend encompassed GPL (most remarkably PE, PC, PI and corresponding lyso-lipids). While these differences were already apparent in the comparison against male lipidomes, they were strongly enhanced by CC medication, but not affected by the endogenous levels of sex hormones in both females (estradiol) and males (testosterone). One interesting practical consequence is that the exact positioning of female subjects along their menstrual cycle might not be required for clinical lipidomics screens.

We argue that CC is a major and often underestimated factor that should be carefully considered when screening lipidomes of females of the reproductive age. The observed differences may mostly reflect the impact of CC, rather than of unfolding metabolic disorders. We are unaware of any published lipidomic screen in which CC-females were either excluded from the study or considered as a separate sub-group. Along the same line, the lipidomics community might need to re-evaluate the possible impact of other life-quality or performance-enhancing medication. While often not considered as drugs and prescribed to otherwise healthy individuals, they may have an unexpectedly strong impact on lipid metabolism.

Plasma lipidomics profiling of healthy male individuals revealed that they were already split into two groups. Although indistinguishable by their anthropometric indices, the smaller group (*ca* 25% of the male cohort) showed a clear trend consistent with unfavourable lipid metabolism, although their clinical indices remained within the acceptable range. We speculate that elevated levels of glycerolipids (TAG and DAG) and CE, along with reduced levels of PE O- and PC O-, relative enrichment of lipid species with polyunsaturated fatty acid moieties, along with unperturbed levels of lyso-lipids (LPE, LPC, LPC O-) are contributing to a collective signature (“lipotype”) of early metabolism disturbance.

It is too early to tell if this compositional trend may have a diagnostic value. These studies should continue on a larger population cohort and over an extended period of time. It would also be important to asses if changes in diet and lifestyle may overt or delay the appearance of first clinically recognizable manifestations of metabolic syndrome among subjects of the risk cluster. However, the very existence of a risk cluster indicates that we might need to reconsider a paradigm of biomarker discovery by focusing on early metabolic trends in otherwise healthy individuals. This should now become increasingly possible since *omics* technologies are able of identifying minor changes in complex molecular composition in any diagnostically promising biological media.

## Material and Methods

### Chemicals

Common chemicals and solvents of ACS or LC–MS grade from Sigma–Aldrich Chemie (Munich, Germany) and methanol (LiChrosolv grade) from Merck (Darmstadt, Germany).

### Cohort recruitment, design and approval of the study

The study was approved by the local competent authority the Ethik-Kommission an der Technischen Universität Dresden (protocol EK328092011). All approached subjects submitted their informed written consent. 103 individuals (48 males; 55 females), all under 33 years of age, were recruited locally. Each participant completed a questionnaire according to http://www.adipositas-portal.de. Clinical and anthropometric indices covered by the medical examination and by a clinical blood test are in Supplementary Table S2. Upon pre-screening completion, 12 males and 20 females did not meet the selection criteria (Supplementary Table S5) and were excluded. The exception was made for 8 out of 36 males (19.4%) and 1 out of 35 females (2.9%) with BMI between 25.0 and 29.9 kg/m^2^ (overweight), because their blood test results were within the requested range.

### Contraception status of female subjects

Out of 35 female participants, 19 used hormonal CC. Out of these 19 CC-females, 4 and 15 used hormonal vaginal rings and oral medication, respectively. Within the latter group, 6 persons were taking daily ethinylestradiol (0.02–0.035 mg)/drospirenone (3 mg); 3 persons: ethinylestradiol (0.03 mg)/dienogest (2 mg); 2 persons: ethinylestradiol (0.03 mg)/levonorgestrel (0.125 mg); 1 person: ethinylestradiol (0.035 mg)/cyproteronacetate (2 mg); 1 person: perethinylestradiol (0.02 mg)/desogestrel (0.15 mg); 1 person: dienogest (2 mg).

### Blood plasma samples collection

Human blood plasma samples were collected in accordance to ethical guidelines and approved standard clinical protocol after overnight fasting. EDTA-plasma was prepared by 10 min centrifugation at 4 °C and 3000 g. Upon collection, plasma samples were immediately shock-frozen in liquid nitrogen and stored at −80 °C until analysed.

### Standards for lipid quantification

Synthetic lipid standards were purchased from Avanti Polar Lipids, Inc. (Alabaster, AL, USA) or Sigma–Aldrich Chemie (Munich, Germany). Stocks of internal standards were stored in glass ampoules at −20 °C until used for plasma analysis. Actual concentrations of standard lipids in stocks were independently validated by direct mass spectrometric analysis using “Quantitative LIPID MAPS standards” (QLMS): high quality reference stock solutions produced, aliquoted, quantified and shipped in sealed glass vials by Avanti Polar Lipids. QLMS validation covered TAG, diacylglycerols (DAG), PC, PE, PI, lyso-phospatidylcholines (LPC), Cer, sphingomyelins (SM).

### Lipid extraction for shotgun analyses

Frozen plasma samples were thawed and lipids were extracted with methyl-*tert*-butyl ether (MTBE) as described^41^. Briefly, 5 μl of EDTA plasma was placed in a 2 ml vial (Eppendorf, Hamburg, Germany). Then, we added 700 μl of a mixture of internal standards in MTBE/methanol (5:1.5; v/v) containing: 6199 pmol of CholE 12:0; 4743 pmol of CholD7; 1720 pmol of TAG 36:0; 366 pmol of DAG 24:0; 165 pmol of Cer 30:1:1; 1987 pmol of PC 25:0; 272 pmol of PE 25:0; 195 pmol of PI 32:0; 712 pmol of SM 30:1:1, 440 pmol of LPC 12:0; 487 pmol of LPC 13:0; 425 pmol of LPE 13:0; and 308 pmol LPE 14:0.

The samples were vortexed at 4 °C for 1 h. Then, 140 μl of water were added, and the tubes were thoroughly vortexed for 15 min at 4 °C. After centrifuging for 15 min at 13,400 rpm on a Minispin centrifuge (Eppendorf, Hamburg, Germany), the upper organic phases were transferred into glass vials and measured the same day. 10 μl of total lipid extract were finally diluted in 90 μl isopropanol/methanol/chloroform 4:2:1 (v/v/v) containing 7.5 mM ammonium formate and used for mass spectrometric analysis.

### Lipid quantification by shotgun mass spectrometry

Mass spectrometric analyses were performed on a Q Exactive instrument (Thermo Fisher Scientific, Bremen, Germany) equipped with a robotic nanoflow ion source TriVersa NanoMate (Advion BioSciences, Ithaca NY, USA) using nanoelectrospray chips with the diameter of spraying nozzles of 4.1 μm. The ion source was controlled by the Chipsoft 8.3.1 software (Advion BioSciences). Ionization voltage was +0.96 kV in positive and −0.96 kV in negative mode; backpressure was set at 1.25 psi in both modes by polarity switching^41^. The temperature of the ion transfer capillary was 200 °C; S-lens RF level was set to 50%. Each sample was analyzed for 5.7 min. FTMS spectra were acquired within the range of *m/z* 400–1000 from 0.02 min to 1.5 min in positive and within the range of *m/z* 400–1000 from 4.2 min to 5.7 min in negative mode at a mass resolution of R_*m/z*_200 = 140000 and automated gain control (AGC) of 10^6^. Free cholesterol was quantified by targeted FT MS/MS within 1.5–4.0 min runtime. For FT MS/MS micro scans were set to 1; isolation window to 0.8 Da; normalized collision energy to 12.5%, AGC to 5 × 10^4^. Lipids were identified by LipidXplorer software^42^. Molecular Fragmentation Query Language (MFQL) queries were compiled for PC, PC O-, LPC, LPC O-, PE, PE O-, LPE, PI, SM, TAG, DAG, Cer, Chol, CE lipid classes and are available at LipidXplorer wiki site: https://wiki.mpi-cbg.de/wiki/lipidx/index.php/Main_Page. The identification relied on accurately determined intact lipid masses (mass accuracy better than 3 ppm). Lipids were quantified by comparing the isotopically corrected abundances of their molecular ions with the abundances of internal standards of the same lipid class. Only lipids whose monoisotopic peaks were detected with a signal-to-noise ratio above the value of 10 were quantified.

### Lipid quantification by LC-MS/MS

Frozen plasma samples were thawed followed by robotic assisted 96-well sample extraction using a Hamilton Microlab Star system (Hamilton Robotics). Simple glycolipids, ranging from hexosylceramide up to Gb3 were extracted from 10 μL plasma as described^43^. Sphingosines and spingosine-1-phosphates were extracted from 25 μL plasma by 1.1 mL of ice-cold methanol containing 0.1% BHT. Gangliosides were extracted from 100 μL plasma as described by^44^ with minor modifications. Eicosanoids were extracted from 150 μL plasma as in^45^. Internal standards were spiked into plasma samples prior extraction. LC-MS/MS analyses were performed on Eksigent XL-100 UHPLC system (AB Sciex) coupled to a QTRAP 5500 triple quadrupole or 6500 QTRAP mass spectrometers (AB Sciex). Hexosylceramides, lactosylceramides and globotriaosylceramides were analysed as described^46^; sphingosines and sphingosine-1-phosphates as in^47^; eicosanoides as in^45^; gangliosides as in^48^. MRM data were processed by MultiQuant software (Sciex).

### Statistical and computational methods

Lipid species quantified in the plasma of less than 6 out of 71 (~10%) study participants were discarded. Mann-Whitney nonparametric test with Benjamini-Hochberg multiple testing correction was used to determine the significance of changes in lipid abundances. To reveal the similarity between multidimensional lipidome compositions the full dataset was analyzed by juxtaposing two complementary unsupervised and parameter-free pattern visualisation techniques: linear dimensionality reduction principal component analysis (PCA) and nonlinear dimensionality reduction non-centred Minimum Curvilinear Embedding (ncMCE)^28^,^29^,^30^. MATLAB and R code for performing ncMCE is available at:

https://sites.google.com/site/carlovittoriocannistraci/5-datasets-and-matlab-code/minimum-curvilinearity-ii-april-2012

### Building the correlation networks

We first compared lipidomics datasets nonCC-females vs males and nonCC-females vs CC-females and determined *p*-values for the abundances of lipid species, lipid classes and also for the clinical variables listed in Supplementary Table 1S and then adjusted them by Benjamini correction. Then only the significant features (with adjusted p-value < 0.05) were selected and then a correlation network was constructed on the significant features, choosing only the significant interactions (with *p*-value < 0.05), which were characterized by a largely significant correlation, i.e. |r| > 0.7, where r is the Pearson correlation coefficient. These steps were repeated in order to construct the networks that represent significant associations between significantly discriminative (across cohorts) molecular and clinical variables.

## Additional Information

**How to cite this article**: Sales, S. *et al*. Gender, Contraceptives and Individual Metabolic Predisposition Shape a Healthy Plasma Lipidome. *Sci. Rep*. **6**, 27710; doi: 10.1038/srep27710 (2016).

## Supplementary Material

Supplementary Information

Supplementary Table S2

Supplementary Table S3

Supplementary Table S4
