# Supplementary material for: Gender, Contraceptives and Individual Metabolic Predisposition Shape a Healthy Plasma Lipidome
Source: Sci Rep. 2016 Jun 14;6:27710. doi: 10.1038/srep27710 (PMC4906355; doi:10.1038/srep27710)
Supplement: Supplementary Information [file srep27710-s1.pdf]

Supplementary Materials

for the manuscript by S.Sales, J.Graessler, S.Ciucci, R.Al-Atrib, T.Vihervaara, K.Schuhmann, D.Kauhanen, M.Sysi-Aho, S.R.Bornstein, M.Bickle, C.V.Cannistraci, K.Ekroos and A.Shevchenko
”Gender, Contraceptives and Individual Metabolic Predisposition Shape a Healthy Plasma Lipidome”

Table of Content

Supplementary Table S1 Basal characteristics of the study cohort (extended table).
Supplementary Table S5 Exclusion criteria and gender-specific causes for exclusion from the study
Supplementary Figure S1: Concentrations of individual lipid species
Supplementary Figure S2 Total concentrations of lipid classes.
Supplementary Figure S3 Values of clinical and anthropometric indices.

Supplementary Tables S2, S3 and **S4** are Excel spreadsheets and uploaded as separate files.

Supplementary Table 1. Basal characteristics of the study cohort (extended table).

		**Men (*n*=36)**			**Women (*n*=35)**			
**Parameter**	**units**	**Mean**	**SD**	**95% CI**	**Mean**	**SD**	**95% CI**	**Mann-WhitneyTest Benjamini corrected (*p*)**
Age	years	24.0	2.7	23.1-24.9	25.5	2.8	24.5-26.4	0.0244
Weight	kg	77.2	8.8	74.2-80.1	61.6	7.9	58.9-64.3	3.418E-08
Height	m	1.82	0.06	1.80-1.84	1.70	0.06	1.68-1.72	3.756E-08
BMI	Kg/m²	23.4	2.4	22.6-24.2	21.3	2.2	20.6-22.0	0.001
Waist	cm	80.6	8.2	77.8-83.3	69.3	6.6	67.0-71.6	4.569E-08
Hip	cm	101.8	6.4	99.6-103.9	100.2	6.1	98.0-102.3	0.1374
Waist-to-hip ratio		0.79	0.05	0.77-0.81	0.69	0.06	0.67-0.71	5.021E-09
Systolic blood pressure	mmHg	122.5	9.4	119.3-125.7	113.4	8.7	110.4-116.4	0.0003
Diastolic blood pressure	mmHg	69.9	7.2	67.4-72.3	65.4	6.1	63.3-67.5	0.009
Triglycerides	mmol/L	0.78	0.27	0.69-0.87	0.83	0.26	0.74-0.92	0.205
Total cholesterol	mmol/L	4.23	0.55	4.04-4.41	4.57	0.47	4.41-4.73	0.205
LDL-cholesterol	mmol/L	2.44	0.54	2.25-2.62	2.40	0.49	2.23-2.57	0.377
HDL-cholesterol	mmol/L	1.55	0.31	1.45-1.66	1.93	0.31	1.83-2.04	2.262E-05
Hemoglobin	mmol/L	9.47	0.60	9.27-9.68	8.34	0.51	8.16-8.51	7.106E-09
Hematocrit		0.44	0.02	0.43-0.45	0.39	0.02	0.39-0.40	2.9E-09
Leukocytes	GPt/L	5.50	1.03	5.15-5.84	5.57	1.20	5.16-5.98	0.384
Platelets	GPt/L	216.4	45.1	201.2-231.7	245.8	49.5	228.8-262.8	0.009
Erythrocytes	TPt/L	5.05	0.25	4.97-5.14	4.49	0.26	4.40-4.58	7.859E-09
Creatinine	µmol/L	84.1	10.4	80.6-87.7	71.7	6.9	69.3-74.0	1.37E-06
Alanine aminotransaminase	µmol/L*s	0.37	0.14	0.32-0.41	0.25	0.10	0.22-0.29	0.0002
Gamma-glutamyltransferase	µmol/L*s	0.30	0.11	0.26-0.34	0.27	0.13	0.22-0.31	0.029
Uric acid	µmol/L	334.5	59.4	314.4-354.6	233.8	36.7	221.2-246.4	2.9E-09
C-reactive protein	mg/L	0.65	0.75	0.39-0.90	1.33	1.49	0.81-1.84	0.055
Hemoglobin A1c	%	5.17	0.31	5.07-5.28	5.21	0.21	5.14-5.28	0.328
Plasma glucose	mmol/L	4.54	0.36	4.42-4.66	4.49	0.30	4.39-4.60	0.282
Free fatty acids	mmol/L	0.31	0.16	0.26-0.37	0.37	0.19	0.30-0.43	0.137
Proinsulin	pmol/L	2.10	1.54	1.58-2.62	2.15	1.84	1.51-2.78	0.386
C-peptide	pmol/L	548.9	216.3	475.7-622.1	597.7	190.9	532.2-663.3	0.116
Insulin	pmol/L	48.3	26.6	39.3-57.3	58.3	40.8	44.3-72.3	0.115
Thyroid-stimulating-hormone	mU/L	2.84	1.81	2.22-3.45	2.60	1.15	2.21-2.99	0.382
Luteinizing hormone	U/L	3.37	1.12	2.99-3.75	3.16	2.77	2.21-4.11	0.054
Follicle-stimulating hormone	U/L	2.76	1.62	2.21-3.31	3.83	3.11	2.76-4.90	0.12
Cortisol	nmol/L	433.5	116.7	394.0-472.9	446.0	156.6	392.2-499.8	0.367
Dehydroepiandrosterone	nmol/L	26.5	10.2	23.0-29.9	24.5	12.3	20.3-28.8	0.176
Sex hormone-binding globulin	nmol/L	22.4	7.4	19.9-24.9	94.0	66.8	71.1-117.0	5.021E-09

Supplementary Table S5.
Exclusion criteria and gender-specific causes for exclusion from the study

**Parameter**	**units**	**Cut-off values**	**Excluded Individuals (n)**	
			Males (12)	***Females (20)***
Blood pressure (systolic/diastolic)	mmHg	> 140 / 85		
ALAT	µmol/L*s	> 0.80		
γGT	µmol/L*s	> 0.80		
Serum creatinine	µmol/L	> 110 men / > 95 women		
C-reactive protein	mg/L	> 5	**3**	**2**
Hemoglobin	mmol/L	< 8.0 men / < 7.4 women		
HbA_{1C}	%	> 5.7		
Total cholesterol[1]	mmol/L	> 5.2	**5**	**14**
LDL-cholesterol[1]	mmol/L	> 4.0		
HDL-cholesterol[1]	mmol/L	< 0.9 men / < 1.1women		
Triglycerides[1]	mmol/L	> 1.7	**2**	
Plasma glucose[1]	mmol/L	> 5.5		
Plasma insulin[1]	pmol/L	> 250		**1**
Uric acid[1]	µmol/L*s	> 490	**1**	

[1] values after 12h overnight fasting; ALAT: alanine aminotransferase, γGT: gamma glutamyltransferase, HbA_{1C}: glycosylated hemoglobin
One male individual was excluded due to missing data; two females were excluded due to taking iron preparations; one due to lactation

Supplementary Figure S1

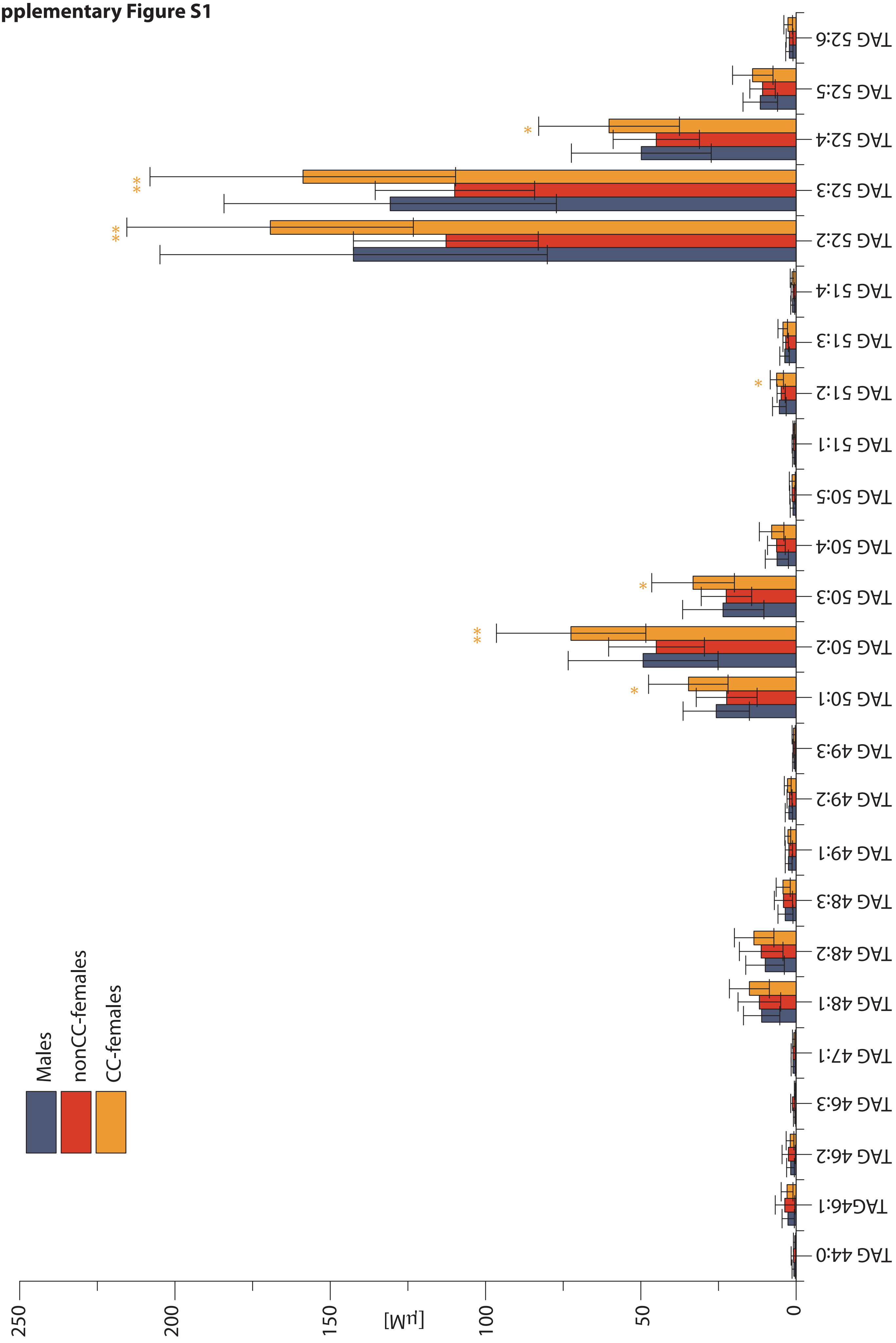

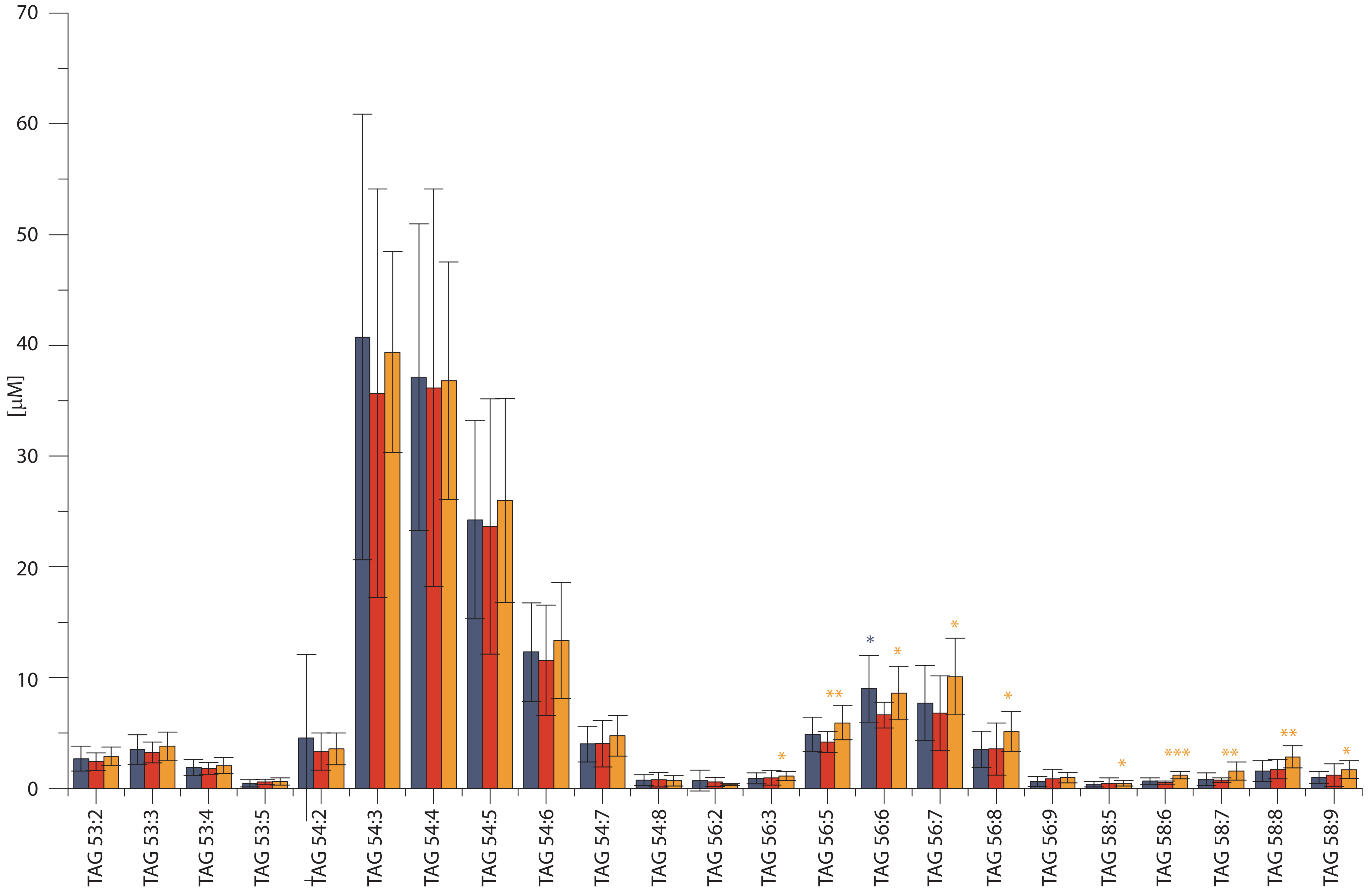

70
60
50
40
30
20
10
0
[µM]
TAG 53:2
TAG 53:3
TAG 53:4
TAG 53:5
TAG 54:2
TAG 54:3
TAG 54:4
TAG 54:5
TAG 54:6
TAG 54:7
TAG 54:8
TAG 56:2
TAG 56:3
TAG 56:5
TAG 56:6
TAG 56:7
TAG 56:8
TAG 56:9
TAG 58:5
TAG 58:6
TAG 58:7
TAG 58:8
TAG 58:9

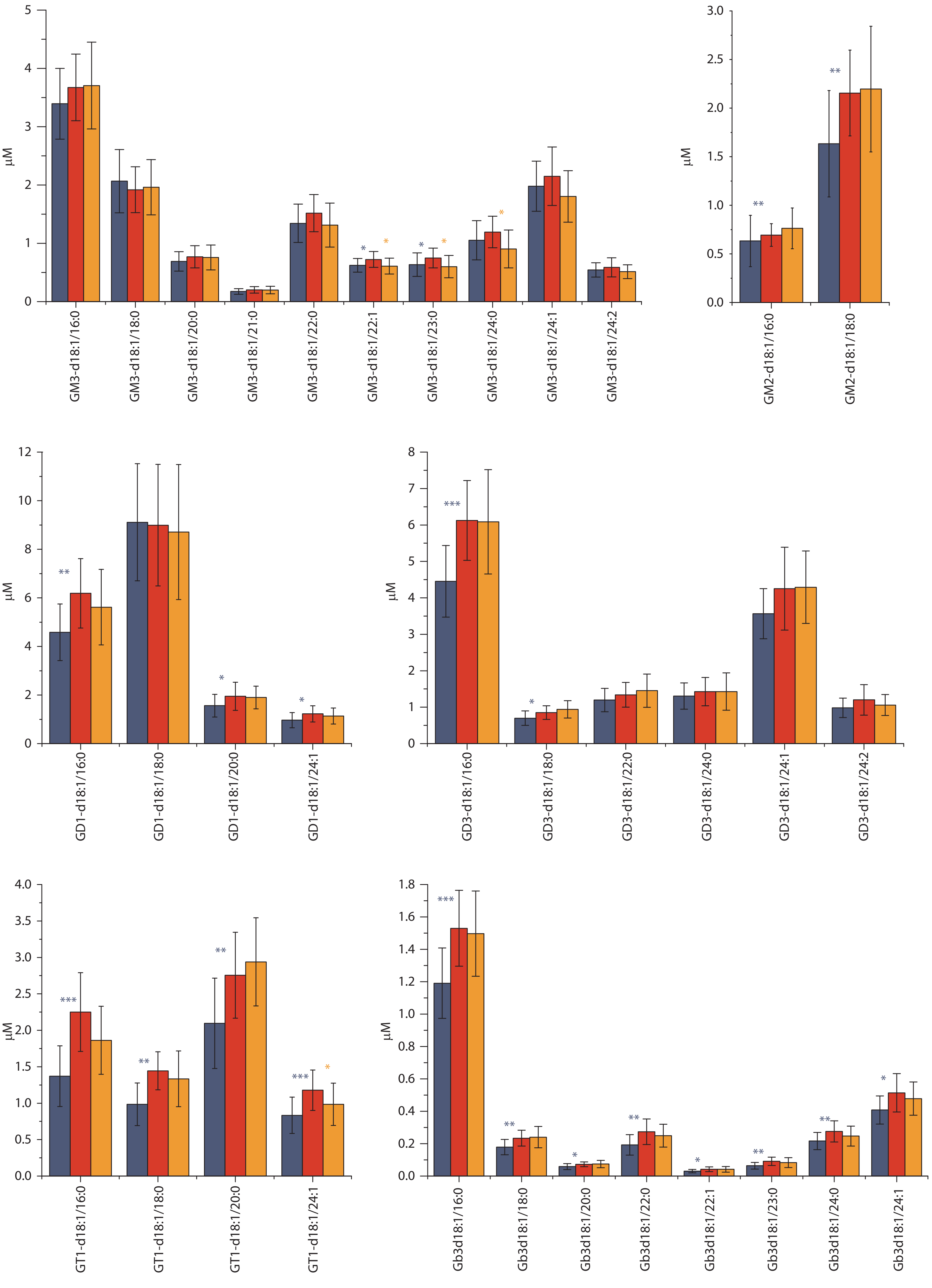
μM
GM3-d18:1/16:0
GM3-d18:1/18:0
GM3-d18:1/20:0
GM3-d18:1/21:0
GM3-d18:1/22:0
GM3-d18:1/22:1
GM3-d18:1/23:0
GM3-d18:1/24:0
GM3-d18:1/24:1
GM3-d18:1/24:2
μM
GM2-d18:1/16:0
GM2-d18:1/18:0
μM
GD1-d18:1/16:0
GD1-d18:1/18:0
GD1-d18:1/20:0
GD1-d18:1/24:1
μM
GD3-d18:1/16:0
GD3-d18:1/18:0
GD3-d18:1/22:0
GD3-d18:1/24:0
GD3-d18:1/24:1
GD3-d18:1/24:2
μM
GT1-d18:1/16:0
GT1-d18:1/18:0
GT1-d18:1/20:0
GT1-d18:1/24:1
μM
Gb3d18:1/16:0
Gb3d18:1/18:0
Gb3d18:1/20:0
Gb3d18:1/22:0
Gb3d18:1/22:1
Gb3d18:1/23:0
Gb3d18:1/24:0
Gb3d18:1/24:1

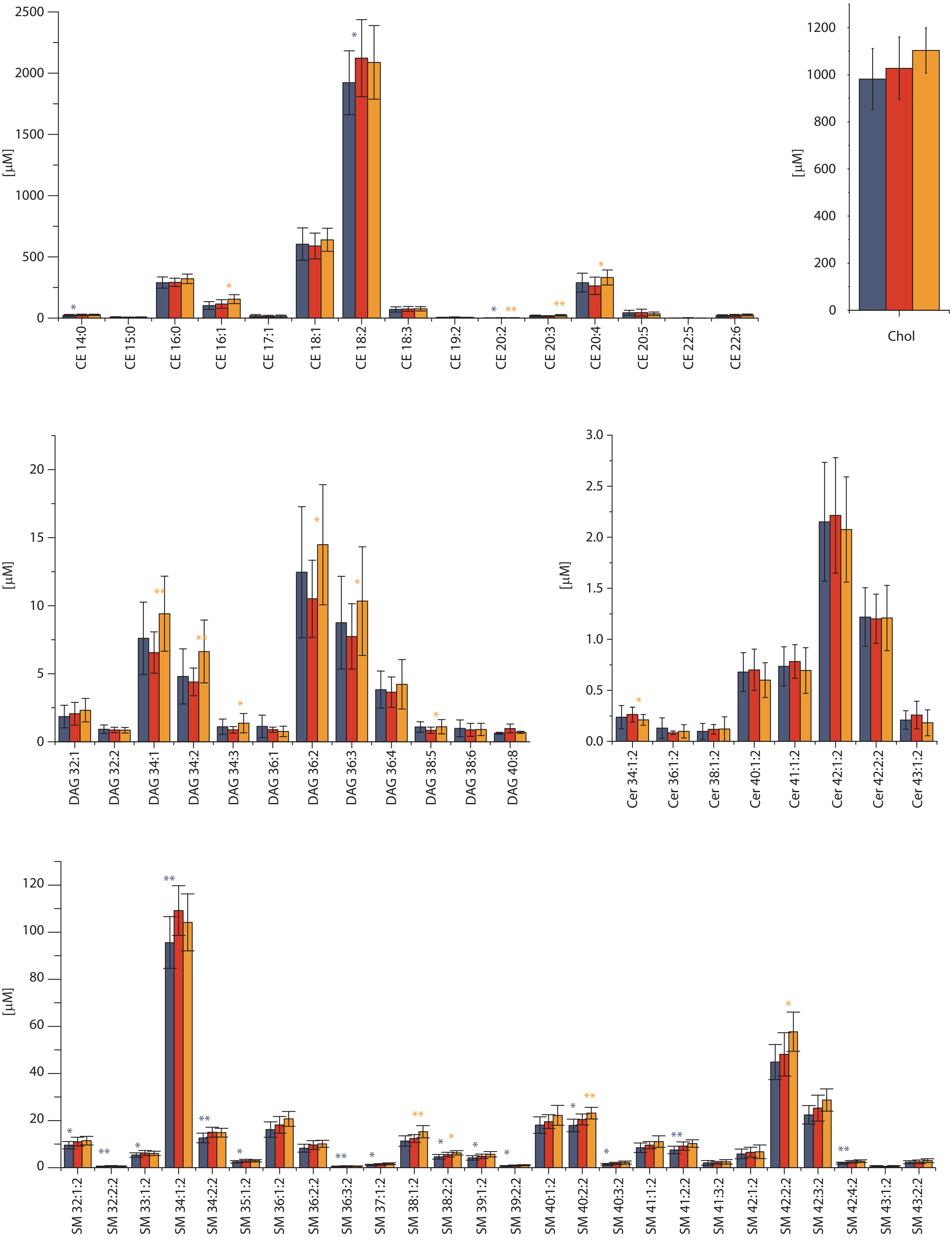

2500
2000
1500
1000
500
0
[µM]
CE 14:0
CE 15:0
CE 16:0
CE 16:1
CE 17:1
CE 18:1
CE 18:2
CE 18:3
CE 19:2
CE 20:2
CE 20:3
CE 20:4
CE 20:5
CE 22:5
CE 22:6
1200
1000
800
600
400
200
0
[µM]
Chol
20
15
10
5
0
[µM]
DAG 32:1
DAG 32:2
DAG 34:1
DAG 34:2
DAG 34:3
DAG 36:1
DAG 36:2
DAG 36:3
DAG 36:4
DAG 38:5
DAG 38:6
DAG 40:8
3.0
2.5
2.0
1.5
1.0
0.5
0.0
[µM]
Cer 34:1:2
Cer 36:1:2
Cer 38:1:2
Cer 40:1:2
Cer 41:1:2
Cer 42:1:2
Cer 42:2:2
Cer 43:1:2
120
100
80
60
40
20
0
[µM]
SM 32:1:2
SM 32:2:2
SM 33:1:2
SM 34:1:2
SM 34:2:2
SM 35:1:2
SM 36:1:2
SM 36:2:2
SM 36:3:2
SM 37:1:2
SM 38:1:2
SM 38:2:2
SM 39:1:2
SM 39:2:2
SM 40:1:2
SM 40:2:2
SM 40:3:2
SM 41:1:2
SM 41:2:2
SM 41:3:2
SM 42:1:2
SM 42:2:2
SM 42:3:2
SM 42:4:2
SM 43:1:2
SM 43:2:2

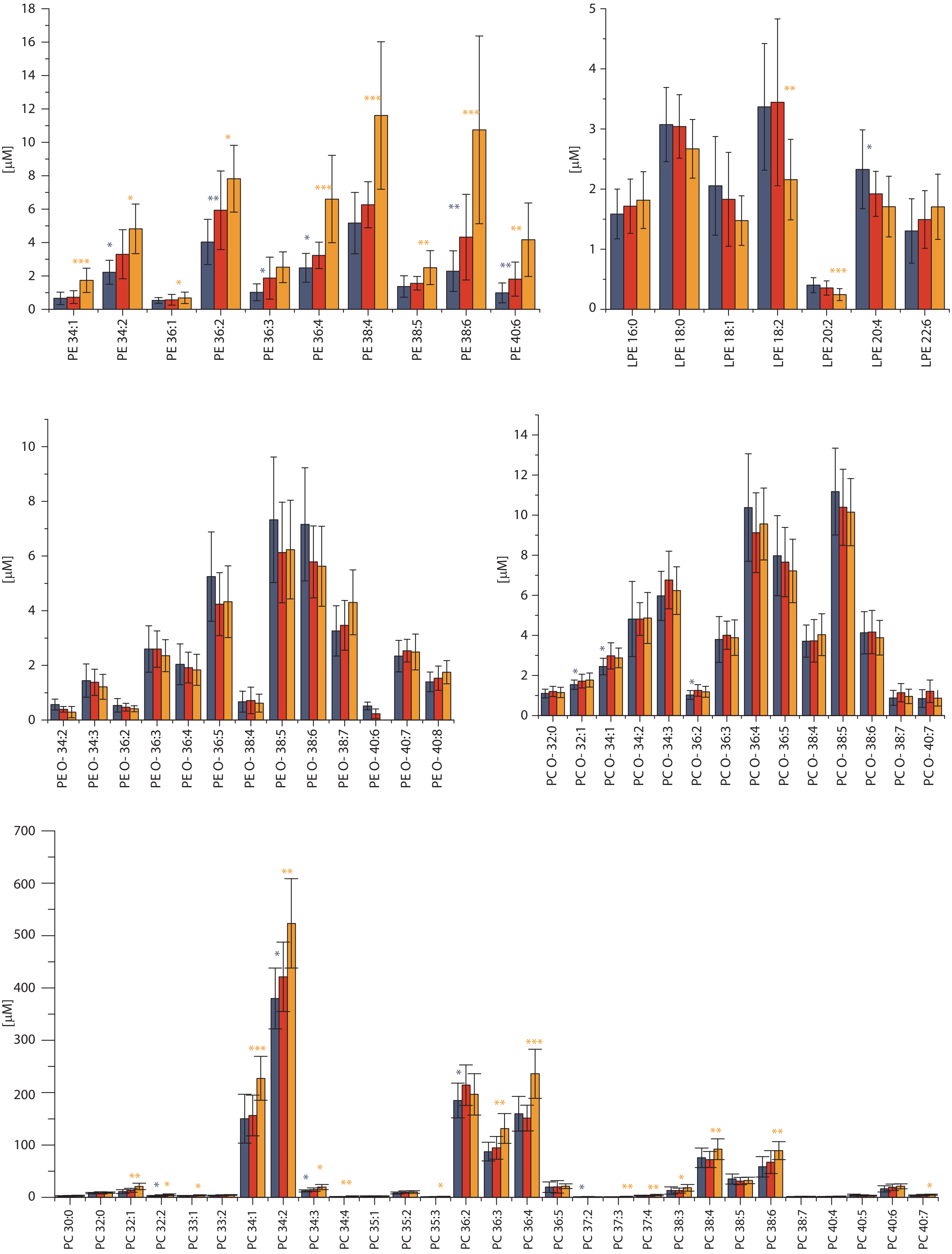

[µM]
PE 34:1
PE 34:2
PE 36:1
PE 36:2
PE 36:3
PE 36:4
PE 38:4
PE 38:5
PE 38:6
PE 40:6
[µM]
LPE 16:0
LPE 18:0
LPE 18:1
LPE 18:2
LPE 20:2
LPE 20:4
LPE 22:6
[µM]
PE O- 34:2
PE O- 34:3
PE O- 36:2
PE O- 36:3
PE O- 36:4
PE O- 36:5
PE O- 38:4
PE O- 38:5
PE O- 38:6
PE O- 38:7
PE O- 40:6
PE O- 40:7
PE O- 40:8
[µM]
PC O- 32:0
PC O- 32:1
PC O- 34:1
PC O- 34:2
PC O- 34:3
PC O- 36:2
PC O- 36:3
PC O- 36:4
PC O- 36:5
PC O- 38:4
PC O- 38:5
PC O- 38:6
PC O- 38:7
PC O- 40:7
[µM]
PC 30:0
PC 32:0
PC 32:1
PC 32:2
PC 33:1
PC 33:2
PC 34:1
PC 34:2
PC 34:3
PC 34:4
PC 35:1
PC 35:2
PC 35:3
PC 36:2
PC 36:3
PC 36:4
PC 36:5
PC 37:2
PC 37:3
PC 37:4
PC 38:3
PC 38:4
PC 38:5
PC 38:6
PC 38:7
PC 40:4
PC 40:5
PC 40:6
PC 40:7

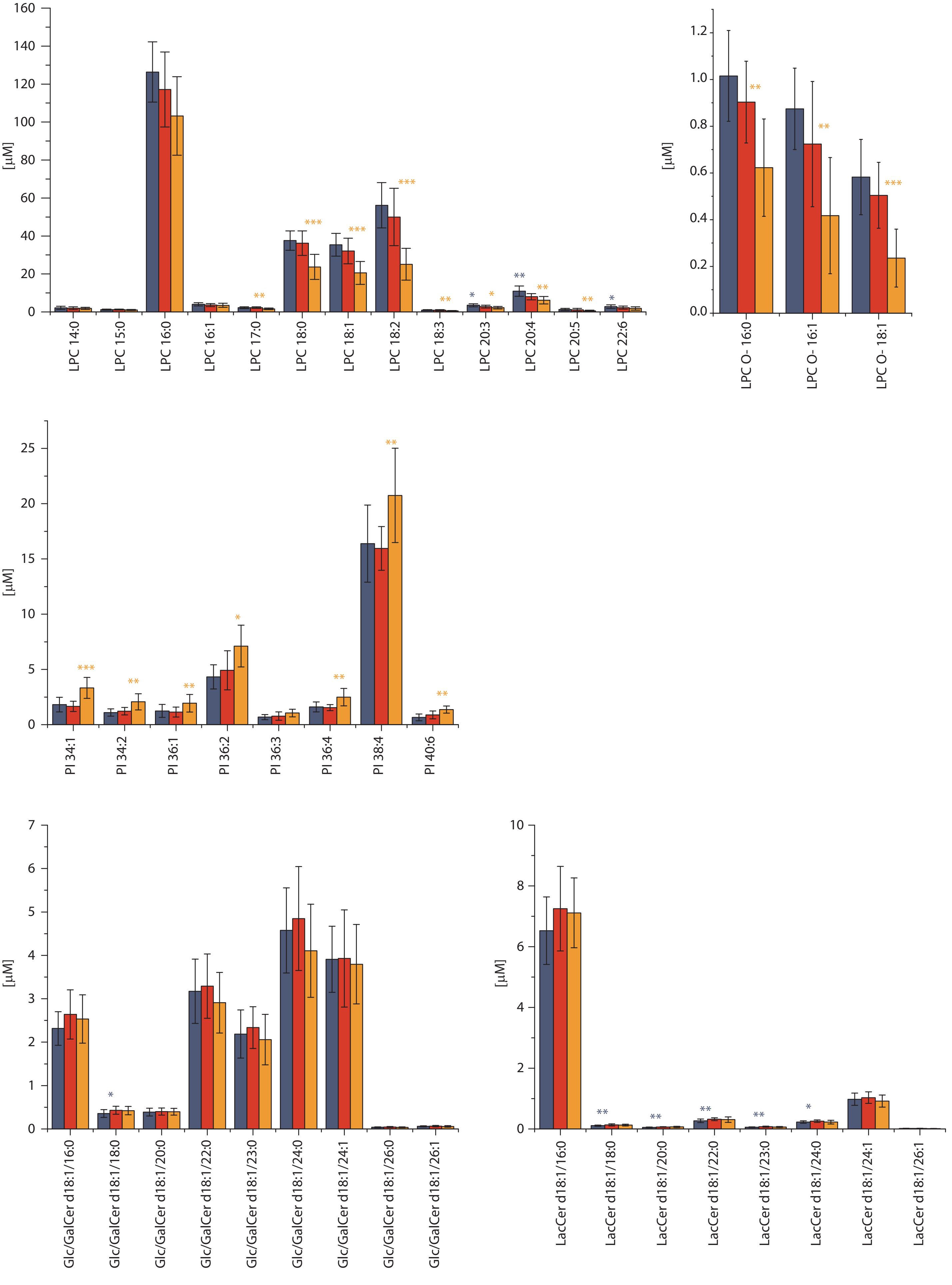

[µM]
0
20
40
60
80
100
120
140
160
LPC 14:0
LPC 15:0
LPC 16:0
LPC 16:1
LPC 17:0
LPC 18:0
LPC 18:1
LPC 18:2
LPC 18:3
LPC 20:3
LPC 20:4
LPC 20:5
LPC 22:6
[µM]
0.0
0.2
0.4
0.6
0.8
1.0
1.2
LPC O- 16:0
LPC O- 16:1
LPC O- 18:1
[µM]
0
5
10
15
20
25
PI 34:1
PI 34:2
PI 36:1
PI 36:2
PI 36:3
PI 36:4
PI 38:4
PI 40:6
[µM]
0
1
2
3
4
5
6
7
Glc/GalCer d18:1/16:0
Glc/GalCer d18:1/18:0
Glc/GalCer d18:1/20:0
Glc/GalCer d18:1/22:0
Glc/GalCer d18:1/23:0
Glc/GalCer d18:1/24:0
Glc/GalCer d18:1/24:1
Glc/GalCer d18:1/26:0
Glc/GalCer d18:1/26:1
[µM]
0
2
4
6
8
10
LacCer d18:1/16:0
LacCer d18:1/18:0
LacCer d18:1/20:0
LacCer d18:1/22:0
LacCer d18:1/23:0
LacCer d18:1/24:0
LacCer d18:1/24:1
LacCer d18:1/26:1

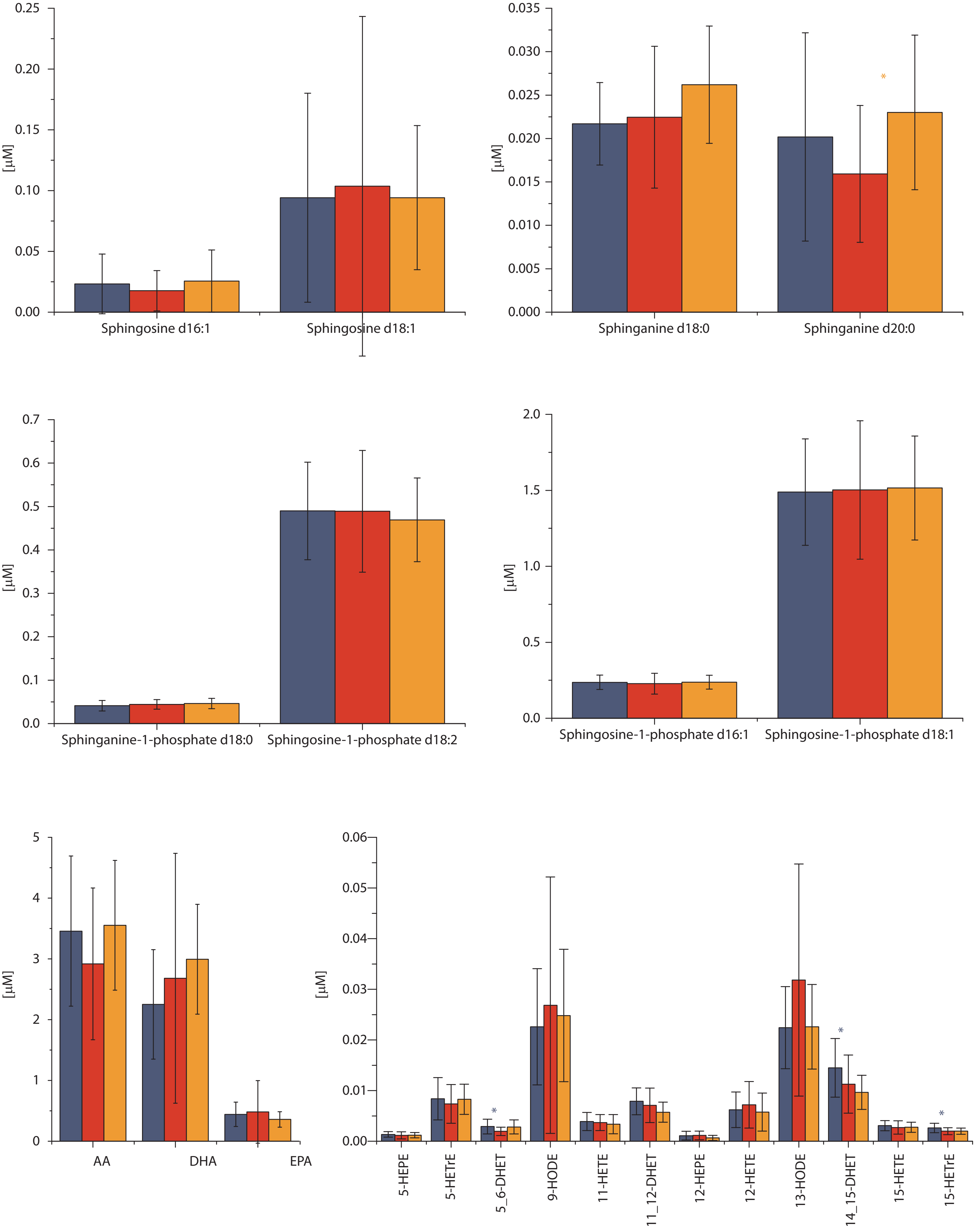

Supplementary Figure S1. Concentrations of individual lipid species. Comparison between males (*n*=36), and nonCC-females (*n*=16) (blue stars), and between nonCC-females (*n*=16) and CC-females (*n*=19) (orange stars) . Data are the mean ± SD. Statistic was performed by nonparametric Mann-Whitney test; Benjamini corrected. *$p<0.05$; **$p<0.01$; ***$p<0.001$.

Supplementary Figure S2

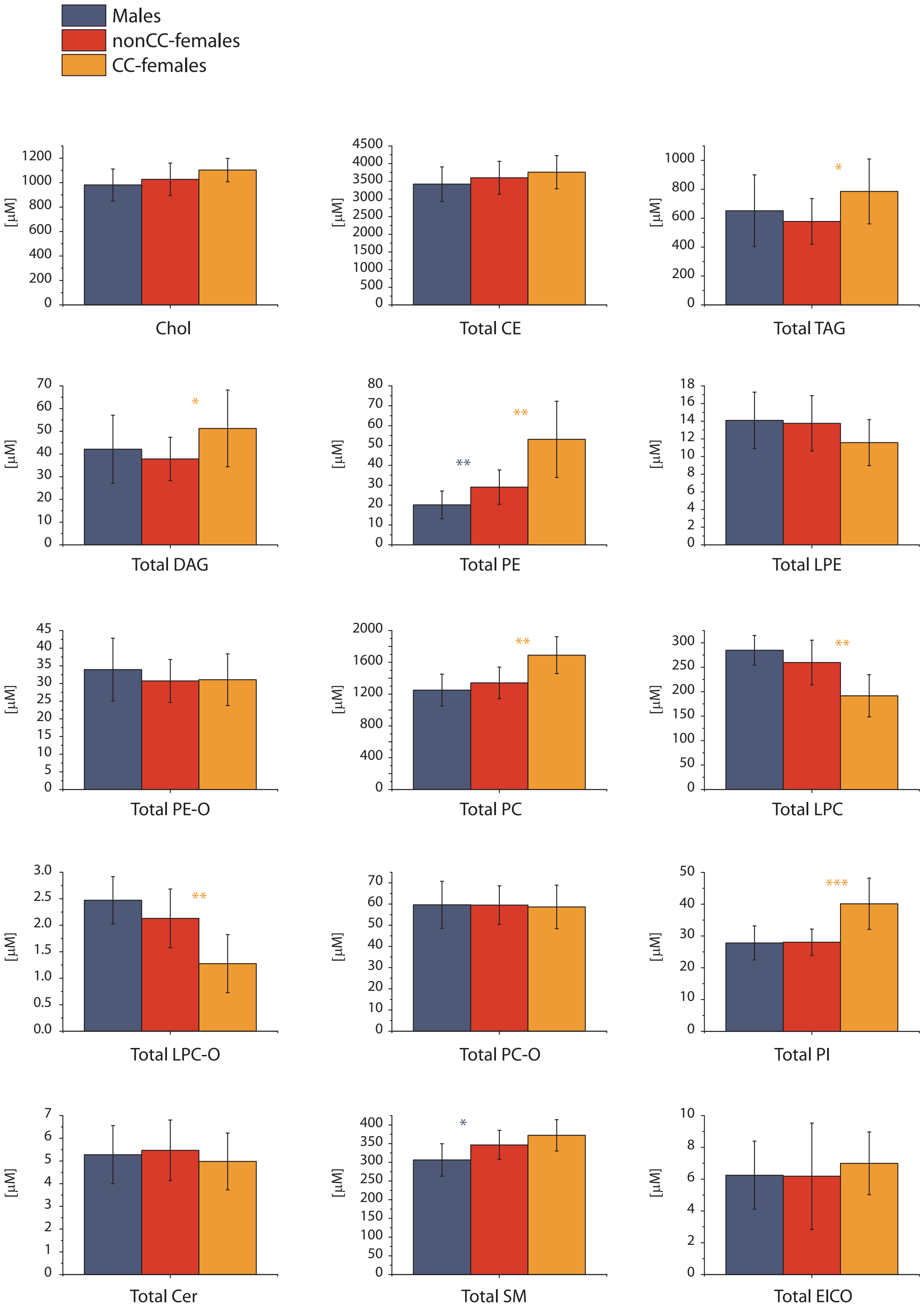

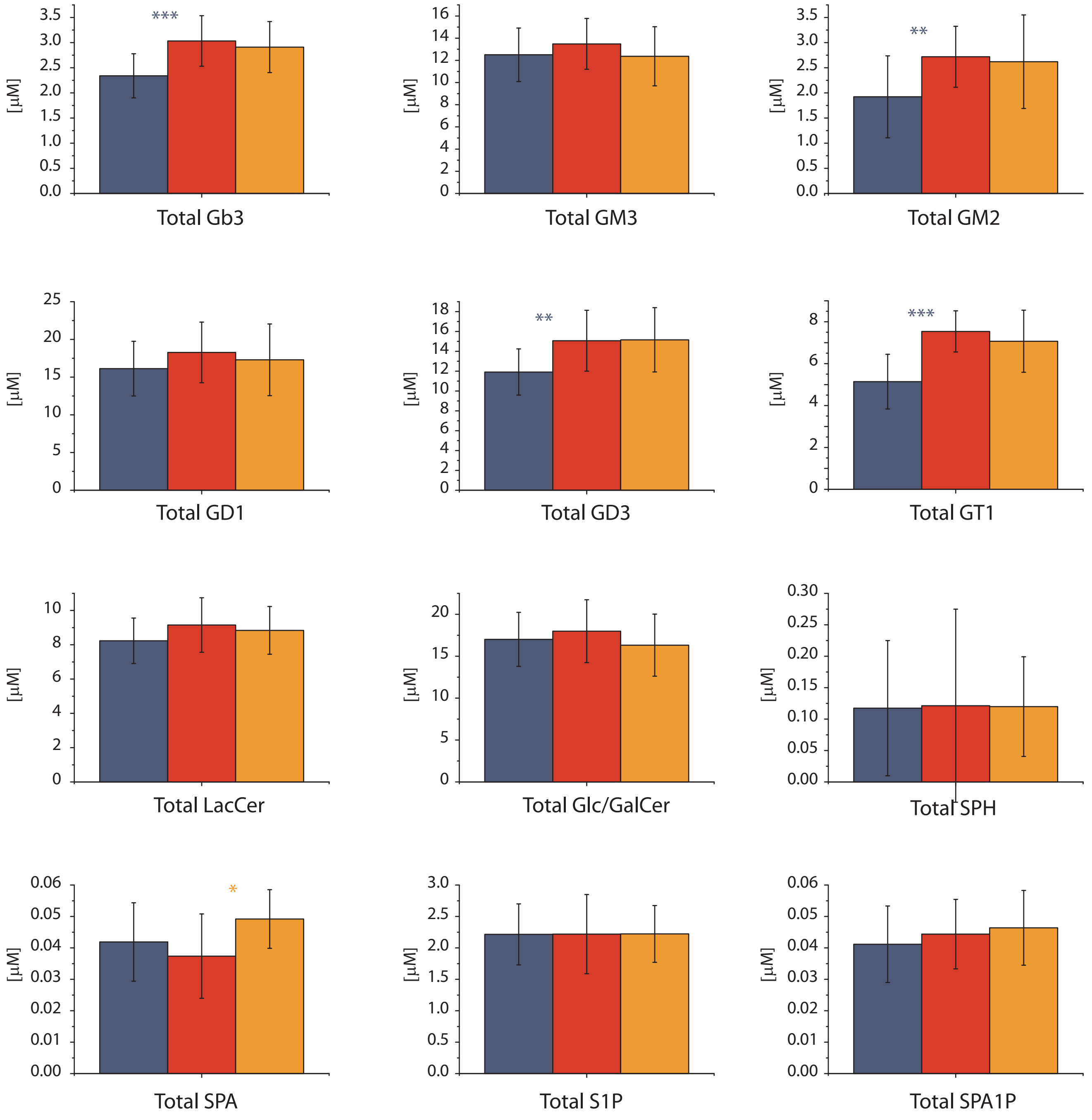

Supplementary Figure S2. Total concentrations of lipid classes. Comparison between males (*n*=36), and nonCC-females (*n*=16) (blue stars), and between nonCC-females (*n*=16) and CC-females (*n*=19) (orange stars) . Data are the mean ± SD. Statistics was performed by nonparametric Mann-Whitney test; Benjamini corrected. *$p<0.05$; **$p<0.01$; ***$p<0.001$.

Supplementary Figure S3

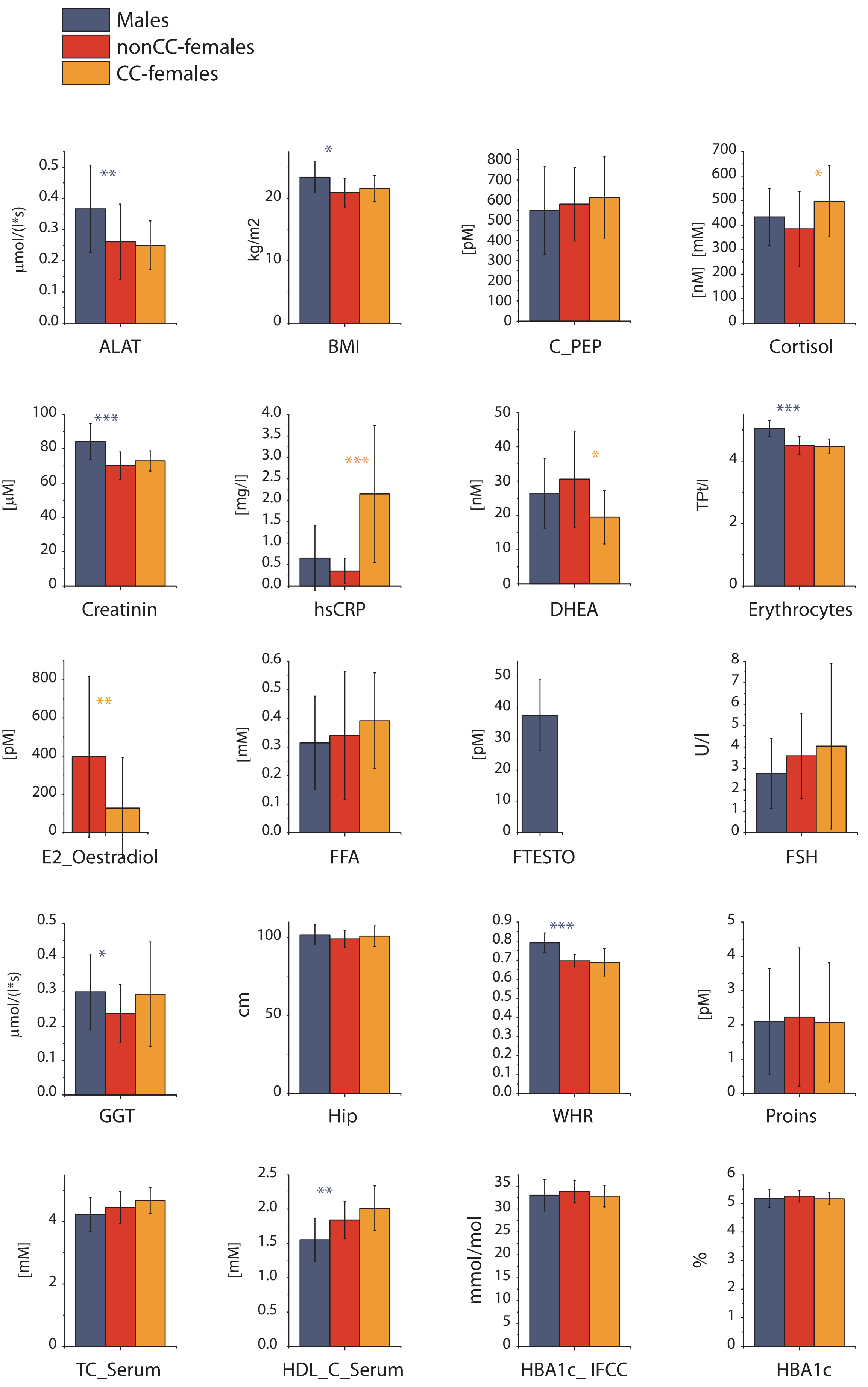

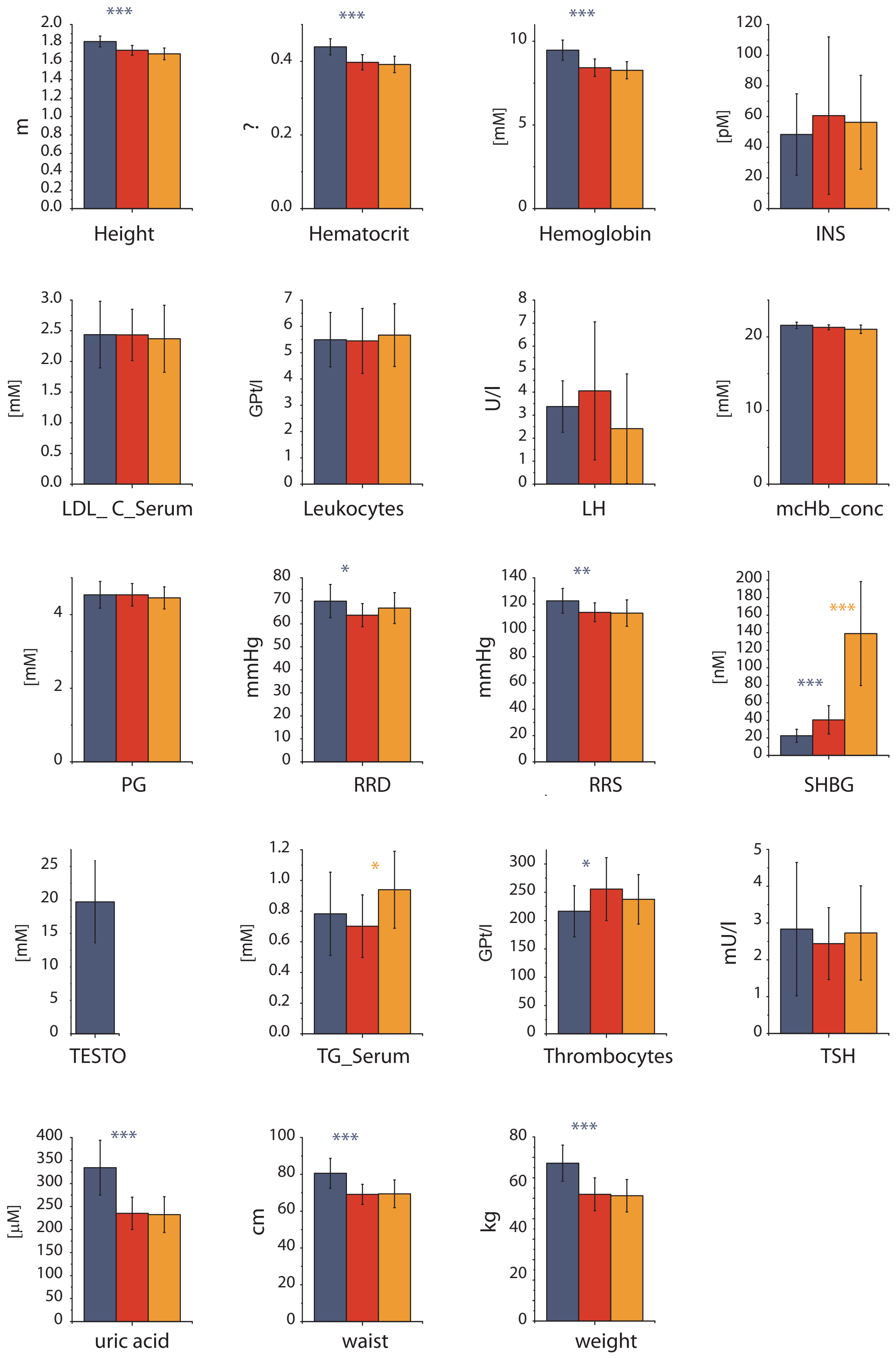

Supplementary Figure S3. Values of clinical and anthropometric indices. Comparison between males (n=36), and nonCC-females (n=16) (blue stars), and between nonCC-females (n=16) and CC-females (n=19) (orange stars) . Data are the mean ± SD. Statistic was performed by nonparametric Mann-Whitney test; Benjamini corrected. *p<0.05; **p<0.01; ***p<0.001.

Supplementary Figure S4

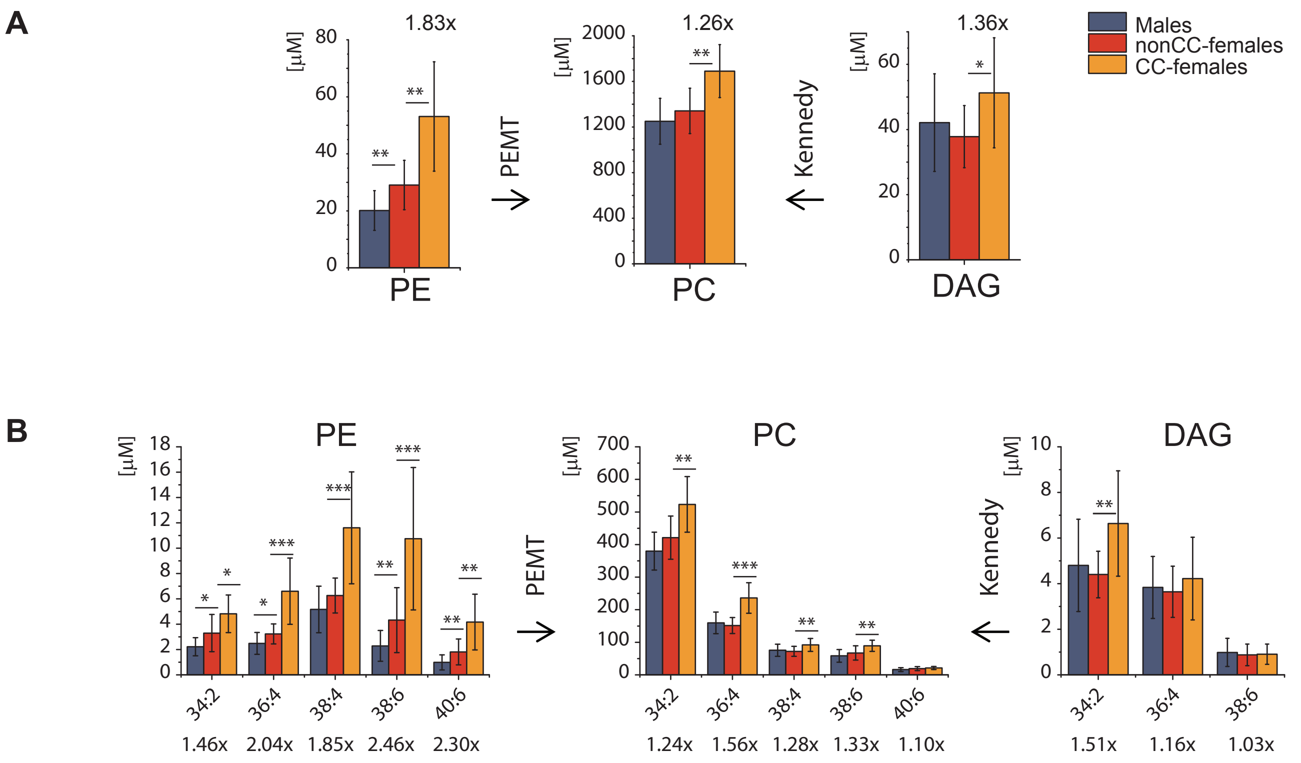

Supplementary Figure S4. Estradiol-dependent and -independent regulation of lipid concentrations in plasma. (A) Total concentration of DAG, PE, and PC in males (*n*=36), nonCC-females (*n*=16), and CC-females (*n*=19). Fold differences between nonCC-females and CC-females are above the concentration bars. **(B)** Several abundat DAG, PE, and PC species and corresponding for differences (shown below concentration bars) between nonCC-females and CC-females. Conversion of polyunsaturated PE species into corresponding PC is faster in nonCC-females compared to CC-females. * $p<0.05$; ** $p<0.01$; $p<0.001$ (nonparametric Mann-Whitney test; Benjamini corrected). Data are the mean ± SD.
